# Non-random distribution of homo-repeats: links with biological functions and human diseases

**DOI:** 10.1038/srep26941

**Published:** 2016-06-03

**Authors:** Michail Yu. Lobanov, Petr Klus, Igor V. Sokolovsky, Gian Gaetano Tartaglia, Oxana V. Galzitskaya

**Affiliations:** 1 Group of Bioinformatics, Institute of Protein Research, Russian Academy of Sciences, 4 Institutskaya str., Pushchino, Moscow Region, 142290, Russia; 2 Bioinformatics and Genomics Programme, Centre for Genomic Regulation (CRG), Dr Aiguader 88, 08003 Barcelona, Spain; 3 Universitat Pompeu Fabra (UPF), 08003 Barcelona, Spain; 4 Institució Catalana de Recerca i Estudis Avançats (ICREA), 23 Passeig Lluís Companys, 08010 Barcelona, Spain

## Abstract

The biological function of multiple repetitions of single amino acids, or homo-repeats, is largely unknown, but their occurrence in proteins has been associated with more than 20 hereditary diseases. Analysing 122 bacterial and eukaryotic genomes, we observed that the number of proteins containing homo-repeats is significantly larger than expected from theoretical estimates. Analysis of statistical significance indicates that the minimal size of homo-repeats varies with amino acid type and proteome. In an attempt to characterize proteins harbouring long homo-repeats, we found that those containing polar or small amino acids S, P, H, E, D, K, Q and N are enriched in structural disorder as well as protein- and RNA-interactions. We observed that E, S, Q, G, L, P, D, A and H homo-repeats are strongly linked with occurrence in human diseases. Moreover, S, E, P, A, Q, D and T homo-repeats are significantly enriched in neuronal proteins associated with autism and other disorders. We release a webserver for further exploration of homo-repeats occurrence in human pathology at http://bioinfo.protres.ru/hradis/.

Eukaryotic and bacterial genomes harbour proteins containing multiple repetitions of specific amino acids called homo-repeats. The functional role of homo-repeats is still unclear^1^, although a tight link with disease exists^2^.

Homo-repeat sizes vary from proteome to proteome^3^,^4^ and are associated with low complexity regions in eukaryotes^5^. Indeed, comparing *H. sapiens* and *E. coli* proteins, we previously reported a significant enrichment of homo-repeats in *H. sapiens*^6^, which can be linked to the presence of structurally disordered regions^7^. Some homo-repeats, such as for instance LLLLLL, occur 11 times more frequently in *H. sapiens* than *D. melanogaster* and *C. elegans*, while TTTTTT are 4 times more abundant in *D. melanogaster* and *C. elegans* than *H. sapiens*^8^. Similarly, the poly-N motif occurs more than 17000 times in 122 proteomes^3^: The NNNNN pattern is connected with *fungi* symbiosis and occurs 21 times in the human proteome^3^. Also HHHHHH repeats are particularly frequent, especially at the N- or C-terminus of polypeptide chains, but their abundance in crystal and NMR structures is often due to biochemical procedures (histidine-tags are useful for purification at a nickel-containing column)^9^. Yet, poly-H are highly frequent in the human proteome and linked to a number of functional roles^6^. For instance, protein kinase DYRK1A (poly-H length of 13) and FAM76B (poly-H length of 10) uses histidine expansions to mediate nuclear speckle trafficking^10^,^11^,^12^.

What functions of poly-repeats are known? For instance, poly-L expansions, especially when located at the N-terminal end of proteins^13^, act as signal peptides and are abundant in membrane proteins^14^. By contrast, poly-Q and poly-A occur more often in transcription factors and poly-K are enriched in a number of metabolic pathways^3^. Similarly, poly-M are connected with voltage-gated calcium channel activity^3^, while poly-P are associated with central nervous system, morphogenesis and through actin cytoskeleton organization, cell morphogenesis, tropomyosin binding and stereocilium^3^. The poly-A tract in the HOXD13 human protein (15 residues in length) is essential for limb development^11^.

Not all homo-repeats are associated with specific roles and investigation of their biological functions is complicated by the widespread occurrence in low-complexity regions of higher eukaryotes^15^. Here, we studied the distribution of homo-repeats in eukaryotic and bacterial proteomes and quantified the difference between expected and real occurrences in 1.5 million sequences. As presence of low complexity regions can cause cellular toxicity by promoting promiscuous interactions^16^, we investigated the relationships between homo-repeat occurrence, number of protein interactions and diseases. We release a dataset at http://bioinfo.protres.ru/hradis/ for further exploration of homo-repeats occurrence in human diseases.

## Results and Discussion

In this study, we focused on the occurrence of homo-repeats in eukaryotic and bacterial proteomes. Previous analyses indicated that homo-repeats of 5 amino acids occur non-randomly^14^,^17^,^18^.

### How large is the difference between the expected occurrences of homo-repeats with real occurrences in 122 proteomes?

How many proteins are expected to contain a homo-repeat of a certain length? If we compute the expected number of proteins <N(M)> harbouring a homo-repeat of M residues in a database containing 1 million protein sequences with average length of 500 residues and uniform amino acid frequency of 1/20, we have:


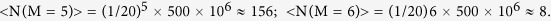


In the case of the human proteome our estimates indicate <N(M = 5)> ≈ 7 and <N(M = 6)> ≈ 0.3.

**Figure 1:**
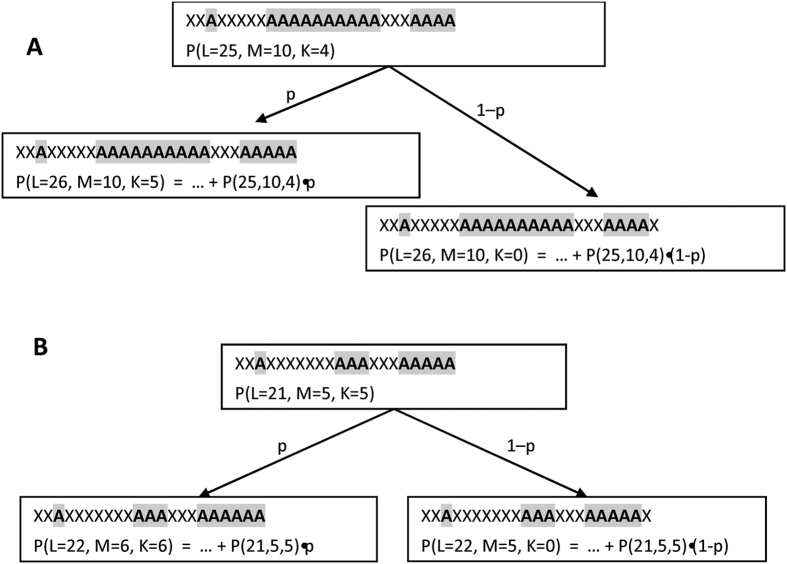
Theoretical estimate of homo-repeat frequencies. Given the length of the sequence (L) and the sizes of the central (M) and C-terminal (K) motifs, it is possible to compute the probability p that a homo-repeat occurs using the recursive formula presented in Eq. 2. (A) The longest homo-repeat is in the central part of the sequence. (**B**) The longest homo-repeat is at the C-terminal.

**Figure 2:**
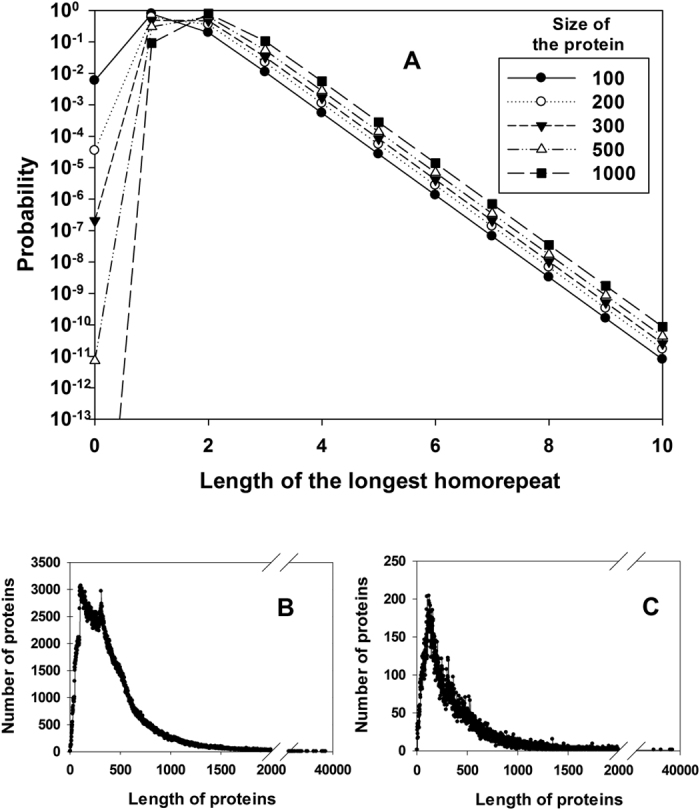
Protein length and expected homo-repeat frequencies. (**A**) Predicted frequencies of the longest homo-repeat at different proteins lengths. Protein length distribution of (**B**) 122 proteomes (average length is 435 ± 425 amino acids) and (**C**) human proteome (average length is 395 ± 530 amino acids).

Can this example be expanded into a more general model to study the occurrence of homo-repeats? To this aim, we have derived a recursive equation (Materials and Methods) that estimates the probability of homo-repeats to occur in the central or terminal parts of a protein sequence (Fig. 1A and Materials and Methods). We used the equation to investigate the frequency of the longest homo-repeat M in a protein sequence of length L (Fig. 1B). Using 122 proteomes (Supplementary Table S1), we studied the length distribution of protein sequences (Fig. 2) and their amino acid frequencies (Supplementary Fig. S1) to measure the expected number of proteins N(M, L) carrying a specific motif [see Materials and Methods, Eq. 1].

**Figure 3:**
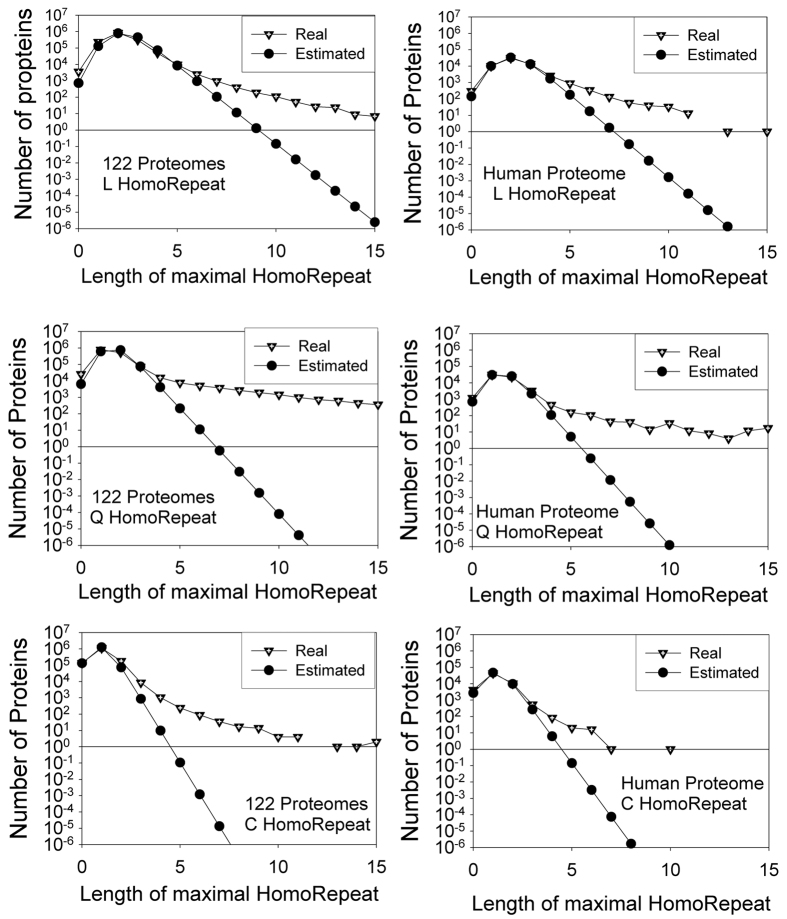
Theoretical vs observed homo-repeat frequencies. For poly-L, poly-Q and poly-C, we report the difference expected and the measured numbers of proteins harbouring the repeats (122 proteomes and human proteome are shown).

The expected frequencies of motif repeats such as poly-Q, poly-L, and poly-C, differ substantially from those observed in real proteomes (Fig. 3; Supplementary Materials): the length of homo-repeats in natural proteomes is much larger than the estimate based on amino acid frequencies and protein length distribution (Fig. 2 and Supplementary Table S1). We report in Table 1 the lengths of homo-repeats whose occurrences in real proteomes have a 10-fold difference from theoretical estimates.

Although previous genome analyses indicated that the minimal homo-repeat length is between 5 and 7 residues^14^,^17^,^18^,^19^, our results indicate the size varies with the amino acid type. For polar and soluble residues^20^ such as H, D, N, K and P, the minimal size is 4, while W, M, Y, F, Q and T, which are often found in amyloid regions^21^, show lengths ≥5. Residues occurring in loops (E, S and G) have lengths ≥5, whereas those containing hydrophobic elements in their side chains (I, R and A) are associated with sizes ≥6 with exception of V and L that have lengths ≥7 and 8. In general, N, D, and K homo-repeats show shorter sizes than for Q, E, and R, although the motif length slightly depends on the kingdom (Table 1). In the case of the human proteome, all the homo-repeats show lengths ≥5 (Table 2), with exception of V, S, A, L, I and M (size: 6) and C (size: 4).

### How many partners do proteins with long homo-repeats have?

**Figure 4:**
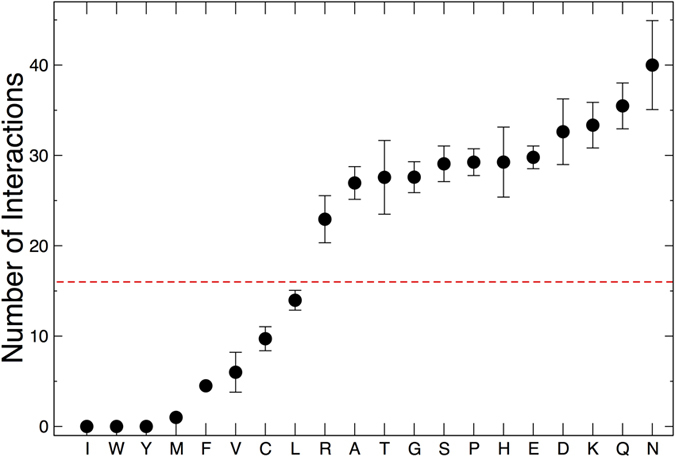
Homo-repeats and protein interactions. Using a total of 94000 physical associations available from BioGRID^22^, we found that human proteins containing poly-E, poly-D, poly-K, poly-Q, and poly-N have more interactions than the rest of the proteome (homo-repeat size is chosen according to Table 2; mean and standard error of the mean are shown). The red line indicates the average number of partners (16 interactions) in *H. sapiens* (total of 320000 interactions).

Our results indicate that homo-repeats are more frequent than expected from theoretical estimates. To investigate what common characteristics have the genes harbouring homo-repeats, we analysed their protein networks using BIOGRID (version 3.4.134)^22^. Using 3514 human proteins carrying homo-repeats with size more than 10 fold larger than expected (Table 2), we found an increase in the number of physical partners of R, A, T, G, S, P, H, E, D, K, Q and N repeats (Fig. 4). Out of 320000 interactions reported in the human proteome, we found that 94000 physical associations involve homo-repeats. The largest number of binding partners was observed for D, K, Q, and N, while I, W and Y are not associated with any interaction (Fig. 4). Thus homo-repeat lengths can be connected with the number of physical associations. While hydrophobic homo-repeats are depleted in partners, hydrophilic ones have a larger number of interactions, which is in agreement with previous literature reporting enrichment of binding partners in polar regions with high structural disorder content^15^,^16^.

### What physico-chemical features define human proteins with many interactions?

**Figure 5:**
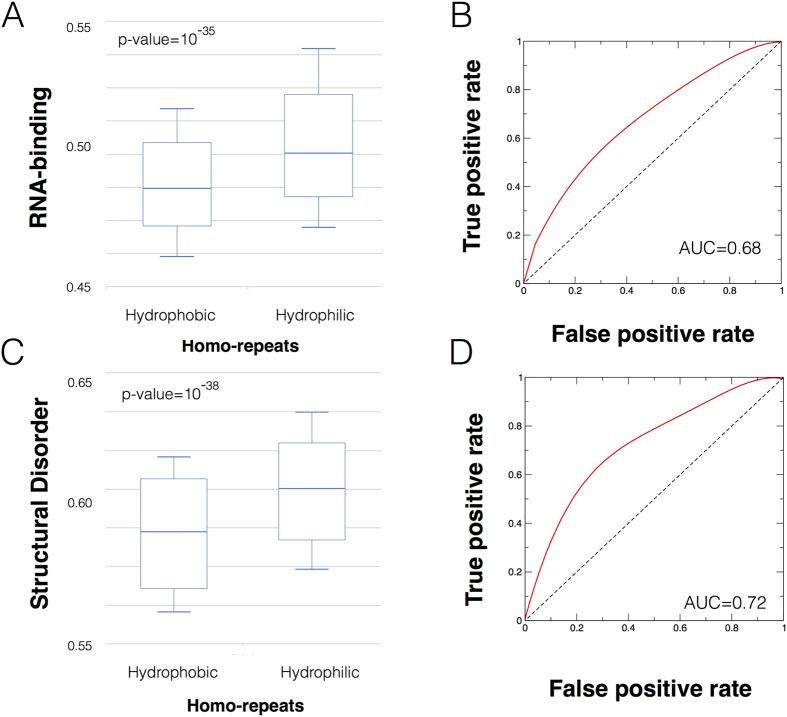
Structural disorder and RNA-binding ability of homo-repeats. Using the *multiclever*Machine approach^23^,^24^, we found that proteins harboring hydrophilic (P, S, N, E, K, R, H, Q and T) homo-repeats are more prone to be RNA-binding^26^ and structurally disordered^25^ than those containing hydrophobic (A, G, C, V, I, L, M, F, Y and W) homo-repeats. (**A**) Box plot analysis (p-value = 10^−35^; Kolmogorov-Smirnov test) and (**B**) Receiver operating characteristic (area under the curve AUC = 0.68) indicate strong enrichments in RNA-binding abilities of hydrophilic homo-repeats. Similar results were observed for structural disorder: (**C**) Box plot (p-value = 10^−38^; Kolmogorov-Smirnov test) and (**B**) Receiver operating characteristic (AUC = 0.72). The analysis, as well as the original datasets can be found at the link http://www.tartaglialab.com/cs_multi/confirm/1358/5f36e6e108/.

**Figure 6:**
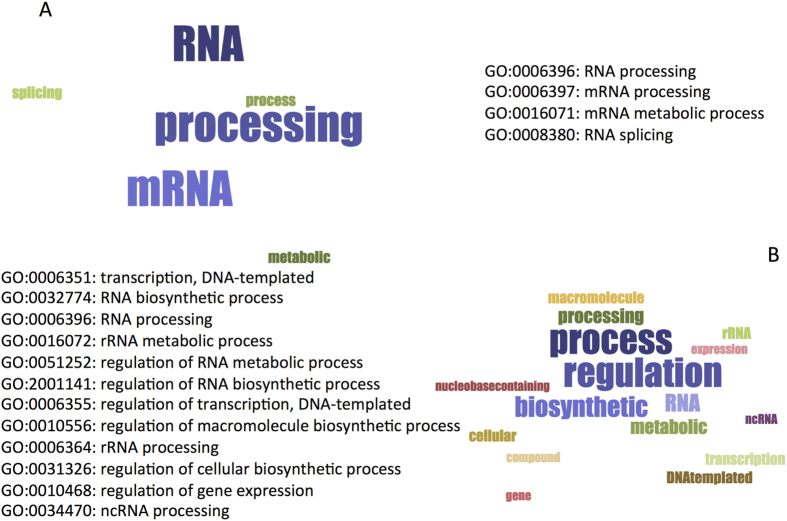
Nucleic acid binding and gene ontology analysis. *multiClever*Machine analysis of AmiGO annotations^51^ indicate that proteins containing poly-R (**A**) and poly-E homo-repeats (**B**) reveal the increase in RNA- and DNA-binding abilities. GO labels are shown together with word-cloud visualization (p-values < 0.01 calculated with Bonferroni’s correction on whole human proteome). The analysis is available at the following links http://www.tartaglialab.com/GO_analyser/render_GO_universal/839/3158792f91/(poly-E) and http://www.tartaglialab.com/GO_analyser/render_GO_universal/840/ea98f8b320/(poly-R).

To understand what physico-chemical features contribute to the interaction ability of homo-repeat proteins, we used the *multiclever*Machine approach^23^,^24^. Based on the consensus of different predictors, *multiclever*Machine identifies signals in protein groups^14^. By directly comparing proteins that contain hydrophobic (A, G, C, V, I, L, M, F, Y and W; total of 1261 proteins) and hydrophilic (P, S, N, E, K, R, H, Q and T; total of 2672 proteins) homo-repeats, we found that the latter are enriched in RNA-binding ability and structural disorder (the analysis is based on homo-repeat sizes reported in Table 2 and is reported at the webserver link http://www.tartaglialab.com/cs_multi/confirm/1358/5f36e6e108/)^25^,^26^. As shown in Fig. 5, the enrichments are significant for both RNA-binding ability (p-value = 10^−35^; Kolmogorov-Smirnov test and area under the ROC curve AUC = 0.68) and structural disorder (p-value = 10^−38^; Kolmogorov-Smirnov test and AUC = 0.72). In agreement with the analysis reported in Fig. 4, proteins with a lower number of interacting partners (i.e., containing homo-repeats with C, F, I, L, M, V, W and Y amino acids) show a decreased amount of structural disorder, while those with a high number of partners (i.e., containing homo-repeats with E, D, G, S, Q, N, K, and H amino acids as well as the intermediate cases R and T) have increased nucleic-acid binding propensity. Thus, our findings are in agreement with previous evidence showing that structural disorder correlates with presence of small and polar amino acids^27^,^28^ and is associated with RNA-binding ability^26^,^29^. Moreover, gene ontology analysis performed with the *multiclever*Machine approach indicates that not only proteins containing poly-R and poly-K (Fig. 6A), but also those with negatively charged homo-repeats are able to bind RNA (Fig. 6A,B), as highlighted by recent studies^30^.

### Relation of homo-repeats to human diseases

**Figure 7:**
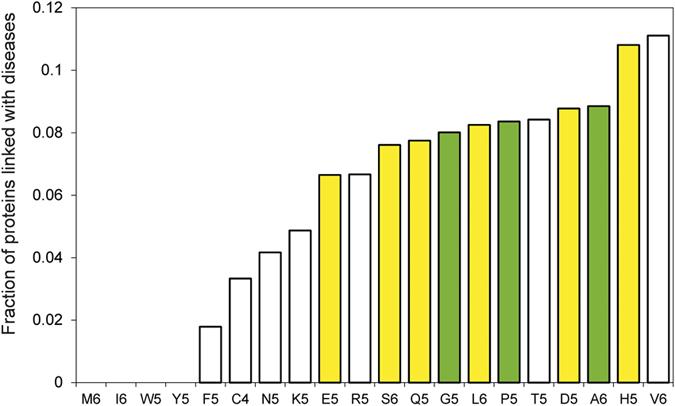
Fraction of proteins linked to disease. Using the OMIM database available at http://www.omim.org/, we found that poly-G, poly-A, and poly-P are strongly associated with disease (standardized Z-score > 5; Material and Methods), followed by poly-E, poly-S, poly-Q, poly-L, poly-D and poly-H. Green colour corresponds to homo-repeats with Z-score > 5, yellow to 3 < Z-score < 5, and white with Z-score < 3 (homo-repeat size is chosen according to Table 2).

In agreement with previous literature data^31^,^32^,^33^,^34^,^35^, we found that Q, G, L, P, T, D, A, H and V homo-repeats have strong propensities to be coupled with pathology (Fig. 7; Table 3; Material and Methods). Indeed, a number of reports indicate that sequences containing repeats such as, for instance, poly-A are associated with diseases, including synpolydactyly type II (gene HOXD13), blepharophimosis (FOXL2), oculopharyngeal muscular dystrophy (PABPN1), infantile spasm syndrome (ARX), and holoprosencephaly (ZIC2)^11^. Similarly poly-Q expansions have been associated with Huntington’s disease, Dentatorubral Pallidolysian Atrophy (DRPLA), and Spinocerebellar Ataxias (SCA)^36^.

Recently, Manuel Irimia and colleagues identified a number of neuron-specific micro-exons (i.e., 27 nt in length) that are switched on during neural differentiation to enhance specific protein-protein interactions. Most of the micro-exon containing proteins are enriched in structurally disordered regions^37^ and about 30% of them are misregulated in the brains of individuals with autism spectrum disorder^37^.

**Figure 8:**
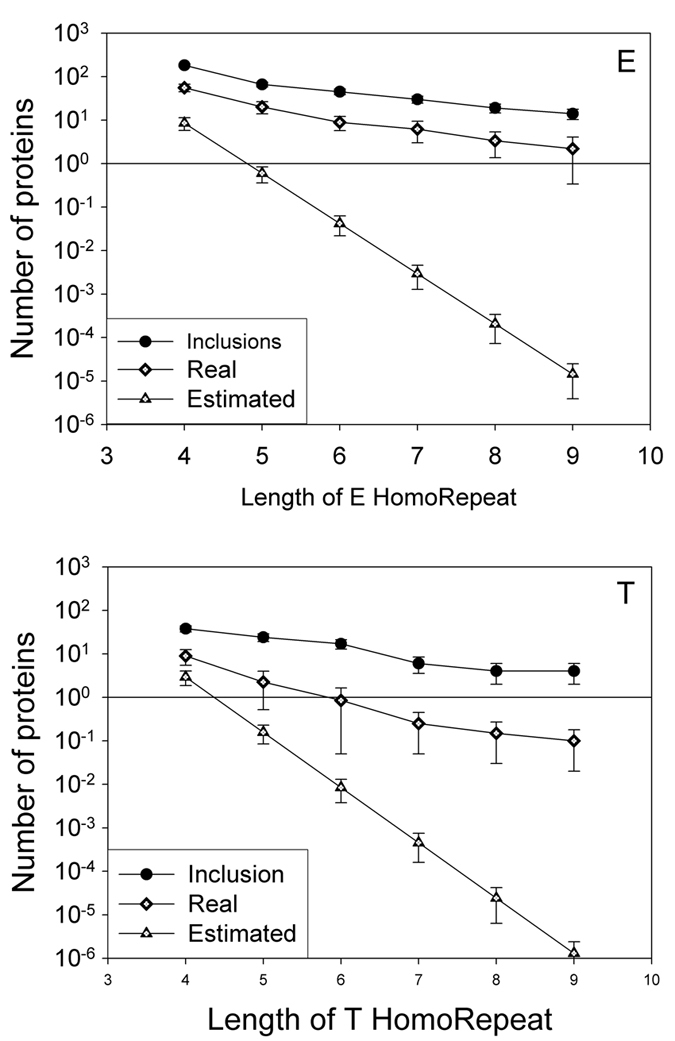
Examples of homo-repeat occurrences in different datasets. For E and T homo-repeats, we report occurrences in protein harbouring micro-exons inclusion (895 neuronal proteins)^37^, human proteome and theoretical estimates based on the occurrence of amino acids in 122 proteomes.

We studied the occurrence of homo-repeats in proteins harbouring micro-exons (895 cases)^37^ comparing their frequencies with expected values calculated on 20 random extractions of the human proteome (Table 4). Increasing the motif length from 4 to 9 amino acids, we found that the following homo-repeats are significantly enriched: 4 – S, E, P, A, Q and T; 5 – S, E, P, A, Q, D and T; 6 – S, E, P, Q, D and T; 7 – S, E, P, Q, T and H; 8 – S, E, P, A, Q, T and H; 9 – S, E, P, Q and T (Table 4; Figs 6 and 8; S, E, P, Q and T are enriched in all considered cases). Interestingly, the enrichments involve polar (D, E, H, Q, S and T) as well as small (A, P and G) amino acids, which can be connected to patterns occurring in proteins with a large number of interactions (i.e., S, P, H, G, D and Q; Fig. 4).

### The HRaDis database

8145 out of 59053 *H. sapiens* proteins (reviewed and un-reviewed entries in the Uniprot database) contain homo-repeats longer than 4 amino acids, which represents a non-negligible component of the proteome (14%). By considering all the homo-repeats currently linked to disease (578 out of 2501 entries; Table 3), the fraction raises to 23%, indicating that homo-repeats are tightly linked with pathology. For instance, out of all the proteins related to neurodegenerative diseases (90 entries), 13 harbour homo-repeats: PERQ2 amino acid-rich with GYF domain-containing protein 2 (PERQ2): poly-Q (sizes: 5, 6, 7, 8 and 9) and poly-K (size: 5); Huntingtin (HD): poly-Q (size: 21) and poly-E (sizes: 5 and 6), poly-P (sizes: 10 and 11); RNA-binding protein FUS (FUS): poly-G (sizes: 7, 10 and 10); Amyloid beta A4 protein (A4) – poly-T (size: 7); Ataxin-2 (ATX2): poly-Q (size: 23); Gap junction gamma-2 protein (CXG2): poly-E (size: 7); Dynactin subunit 1 (DCTN1): poly-A size: 5); NADH-ubiquinone oxidoreductase chain 6 (NU6M): poly-V (size: 5); Pantothenate kinase 2, mitochondrial (PANK2): poly-E (size: 6); Presenilin-2 (PSN2): poly-E (size: 5); Probable helicase senataxin (SETX): poly-D (size: 5); Synphilin-1 (SNCAP): poly-N (size: 5) and Mitochondrial import inner membrane translocase subunit Tim8 A (TIM8A): poly-S (size: 6). This list expands publicly available repositories, such as for instance “PolyQ”^38^, in which only four proteins (ATX1, ATX2, ATX7 and IID) were associated with disease.

To better investigate the link between homo-repeat occurrence and disease, we release the HRaDis database (HomoRepeats and human Diseases, available at http://bioinfo.protres.ru/hradis/), in which human sequences are reported along with OMIM classifications and GO annotations.

## Conclusions

In this work, we showed that the number of homo-repeats in eukaryotic and bacterial proteomes is significantly larger than expected from theoretical estimates. Our calculations indicate that the minimal length that is statistically significant varies with amino acid type and proteome. In *H. sapiens*, occurrence of homo-repeats is associated with high content of structurally disordered regions and enhanced RNA-binding potential, which is in line with recent experimental findings^26^,^29^. We also observed that protein containing homo-repeats have a large number of interactions, which can promote perturbation of protein networks and cause dysfunction^39^.

Although the functional roles of homo-repeats are unknown, we found that their occurrence is associated with pathology. Certain homo-repeats such as for instance the poly-A tract in Homeobox 2B protein (PHOX2B) are highly conserved in vertebrate species and might have biological function. Yet, it has been reported that poly-A is frequently linked with diseases such as synpolydactyly type II (HOXD13), blepharophimosis (FOXL2), oculopharyngeal muscular dystrophy (PABPN1) and infantile spasm syndrome (ARX)^11^. Similarly, poly-Q expansions are associated with neurodegeneration^36^ and their length is proportional to disease severity^40^. The link between homo-repeats and disease is particularly relevant if we consider that a recent study report involvement of low complexity regions in proteins involved in autism^37^.

Possible models for the evolution of homo-repeats have been proposed^41^,^42^,^43^,^44^. Yet, they are still debated, and to assess possible functions, further biological information is necessary. One interesting mechanism that links homo-repeats with protein dysfunction, is that amino acid expansions can be caused by slippage errors in DNA replication, recombination and repair^45^,^46^,^47^,^48^,^49^.

We hope that our work will be useful for the characterization of homo-repeats in the human proteome and that starting from direct analysis of sequences available at http://bioinfo.protres.ru/hradis/, it will be possible to build a catalogue to decipher the biological functions as well as the evolutionary patterns of these sequences.

## Material and Methods

### Probability of occurrence of the longest homo-repeat at different protein lengths

For a polypeptide of length L containing two amino acid types A and X (any amino acid different from A), the probability of finding A in any region of the chain is equal to *p* (the probability of finding X is equal to 1-*p*). Assuming that M is the longest homo-repeat of amino acid A (if A is absent, then M = 0) and K is the length of the homo-repeat adjacent to the C-end of the chain (if the chain terminates with X, then K = 0), we can determine the probability of a homo-repeat in an iterative way. Indeed, if A is added at the C-end, K increases by 1 (if K = M, then M is incremented by 1). The probability of adding A is P(p, M, K + 1, L) = P(p, M, K, L − 1)*p or P(p, M + 1, M + 1, L) = P(p, M, M, L − 1)*p (Fig. 1). By contrast, if X is added, then M does not change, and K becomes 0, and the probability event is P(p, M, 0, L) = P(p, M, K, L − 1)*(1 − p) (Fig. 1). Thus, knowing the joint distribution of M and K for the chain length L–1, it is possible to calculate the distribution of M and K for the chain length L. For a chain with one residue: P(p, M = 0, K = 0, 1) = p and P(p, M = 1, K = 1, 1) = 1 − p (the probability of other M and K values for a chain with one residue is equal to zero). By adding up the values for K (0 ≤ K ≤ M), we calculated the probability depending on the length of the largest homo-repeat M and the chain length L (see Results section).

If we take the distribution lengths of proteins and frequencies from the set of 122 proteomes (see Supplementary Table 1) we can measure the expected number of proteins carrying a specific motif size M:


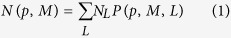


where 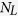 is the number of proteins with length L in the database.

### Calculation of the probability of homo-repeats occurence

If the probability of finding two homo-repeats with length M is small, our Eq. 1 can be approximated (M ≪ L and M ≠ 0). If the homo-repeat lies at the C-term of the protein, there will be M amino acids of type A and another amino acid X with probability of *p*^*M*^(1 − *p*). If the homo-repeat lies in the middle of the protein, there will be M amino acids of type A and two other amino acids at the edges with probability of *p*^*M*^(1 − *p*)^2^. Taken into account that the homo-repeat can be placed in two positions at the edges of the protein and (L − M − 1) in the middle position, the overall homo-repeat probability is:


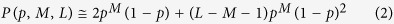


As natural proteins are shorter than 1000 residues, the approximation works at p ≤ 0.05 and M ≥ 4 (*Lp*^*M*^ < 0.01). We note that some amino acids, such as for instance leucine, occur with frequency p ≈ 0.1. In such cases, the approach works well if M ≥ 5.

### Statistical analysis of homo-repeats and link with disease

If homo-repeat and disease frequencies are independent, the distribution has an average number of proteins.


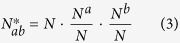


root-mean-square deviation


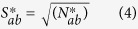


Z-value


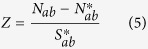


In Eq. 3, 4 and 5, *N* is the number of proteins in the human proteome, 59053. *N*_*a*_ is the number of proteins associated with disease (2501, see Table 3), and *N*_*b*_ is the number of proteins with homo-repeats with the length larger or equal to 5. *N*_*ab*_ is the number of proteins carrying both characters in our database.

### cleverMachine

The cleverMachine (CM) algorithm analyzes physico-chemical properties of two protein datasets^50^. The tool creates profiles, or *physico-chemical signatures*, for each protein, utilizing a large set of features - both experimentally and statistically derived from other tools. In our analysis we used a number of physico-chemical properties (hydrophobicity, alpha-helix, beta-sheet, disorder, burial, aggregation, membrane and nucleic acid-binding propensities) and 10 propensity predictors per feature. Only differentially enriched properties (p-values < 10^−5^ using Fisher’s exact test) were used in the calculations. Further information can be found at http://s.tartaglialab.com/page/clever_suite.

### multiCleverMachine

The *multiclever*Machine extends the concept of binary comparisons (CM) between protein datases by introducing signal and negative sets^23^,^24^. After submission of one or more sets for signal and one or more sets as a negative group, the *multiclever*Machine creates a CM run for each possible combination of elements from the signal and negative sets. The result is presented in an easy-to-read format, allowing at-a-glance interpretation of the CM submission. The *multiclever*Machine provides visualisation of enrichment strengths per group, enabling to see easily for which groups the various properties like disorder, alpha-helical propensity, etc. are enriched. More details about the method are available at http://www.tartaglialab.com/cs_multi/submission. In addition to the visualisation of individual enrichments, *multi*CM links each of the datasets to gene ontology analysis (http://www.tartaglialab.com/GO_analyser/universal and related documentation). To calculate GO enrichments, *multiclever*Machine uses built-in datasets containing all entries available for the proteome of interest (reference set)^23^,^24^.

## Additional Information

**How to cite this article**: Lobanov, M. Y. *et al*. Non-random distribution of homo-repeats: links with biological functions and human diseases. *Sci. Rep.* **6**, 26941; doi: 10.1038/srep26941 (2016).

## Supplementary Material

Supplementary Information

## Figures and Tables

**Table 1:** Lengths of homo-repeats whose frequencies in real proteomes have a 10-fold difference from theoretical estimates.

	C	W	M	H	Y	N	K	F	D	P	Q	I	T	E	S	R	V	G	A	L	N^*^	N^**^
Metazoa	4	5	5	4	5	5	5	5	5	5	5	6	5	5	5	5	6	5	5	6	424	17
Viridiplantae	4	4	5	4	5	5	5	5	5	4	4	6	5	5	5	5	6	5	5	6	108	5
Stramenopiles	4	4	5	4	–	4	4	5	5	4	4	–	5	5	5	5	7	5	6	7	12	1
Choanoflagellida	4	4	4	5	5	5	4	5	5	5	5	6	5	5	6	6	7	6	6	7	9	1
Euglenozoa	4	4	5	4	5	4	4	4	5	5	4	5	5	5	5	6	7	5	5	6	44	4
Alveolata	5	4	5	5	6	6	6	5	5	4	5	8	5	5	5	4	–	4	5	7	50	6
Amoebozoa	4	4	4	4	5	5	5	5	5	4	4	8	5	5	5	5	5	4	4	7	25	2
Diplomonadida	–	–	–	–	–	6	–	–	6	5	5	–	6	6	7	6	5	6	7	–	17	3
Fungi	4	5	5	4	5	5	5	5	5	5	4	7	5	5	6	5	6	5	6	8	551	58
Bacteria	5	–	–	5	–	6	5	6	6	5	5	–	6	7	6	6	8	6	7	9	210	25

N^*^ is the number of proteins (×10^4^), N^**^ is the number of proteomes.

**Table 2:** Lengths of homo-repeats whose occurrence differs at least 10-fold between natural and expected human proteomes.

AA	C	W	M	H	Y	N	K	F	D	P	Q	I	T	E	S	R	V	G	A	L
Length	4	5	6	5	5	5	5	5	5	5	5	6	5	5	6	5	6	5	6	6

**Table 3:** Number of proteins with homo-repeats larger than 4 associated with disease according to the OMIM database http://www.omim.org/ (bold characters correspond to standardized Z-score > 5).

	Proteins	C	M	F	I	L	V	W	Y	A	G	T	S	Q	N	E	D	H	R	K	P
Total	59053	38	3	56	25	1503	49	1	7	1300	836	190	1175	529	24	1625	262	148	270	554	1363
Disease	2501	2	0	1	1	**125**	7	0	0	**105**	**67**	16	**86**	41	1	108	23	16	18	27	**114**

**Table 4:** Homo-repeat enrichments in neuronal proteins harboring micro-exons.

	C (inclusions)						R (proteome)					
	4	5	6	7	8	9	4	5	6	7	8	9
S	**174 ± 13**	**63 ± 8**	**26 ± 5**	**14 ± 4**	**12 ± 3**	**10 ± 3**	**64.5 ± 15.0**	**15.3 ± 6.0**	**6.0 ± 2.4**	**3.1 ± 1.7**	**1.9 ± 1.4**	**1.4 ± 1.0**
L	94** ± **10	36** ± **6	18** ± **4	2±1	–	–	61.2** ± **10.2	18.7** ± **6.0	7.1** ± **3.4	4.0** ± **1.5	1.5** ± **1.0	1.0** ± **1.0
E	**182 ± 13**	**66 ± 8**	**45 ± 7**	**30 ± 5**	**19 ± 4**	**14 ± 4**	**55.9 ± 10.9**	**20.2 ± 6.3**	**8.9 ± 3.2**	**6.2 ± 3.2**	**3.4 ± 2.0**	**2.2 ± 1.9**
P	**136 ± 12**	**67 ± 8**	**39 ± 6**	**28 ± 5**	**22 ± 5**	**13 ± 4**	**50.3 ± 12.7**	**17.8 ± 5.8**	**8.5 ± 2.9**	**5.3 ± 3.2**	**3.0 ± 1.4**	**1.7 ± 1.2**
A	**98 ± 10**	**45 ± 7**	25** ± **5	12** ± **3	**11 ± 3**	4** ± **2	**48.3 ± 8.2**	**15.4 ± 3.6**	8.1** ± **3.4	4.4** ± **1.8	**2.7 ± 1.6**	2.0** ± **1.4
G	75** ± **9	**43 ± 7**	21** ± **5	11** ± **3	5** ± **2	1** ± **1	34.8** ± **8.5	**12.6 ± 4.0**	5.8** ± **2.9	3.7** ± **2.2	1.7** ± **1.1	0.9** ± **1.1
K	47** ± **7	17** ± **4	5** ± **2	1** ± **1	–	–	24.0** ± **7.3	5.8** ± **3.1	2.5** ± **1.5	0.9** ± **1.1	1.0** ± **1.5	0.4** ± **0.6
R	31** ± **6	10** ± **3	–	–	–	–	23.6** ± **4.6	4.9** ± **2.2	1.2** ± **0.9	0.3** ± **0.5	–	0.1** ± **0.3
Q	**42 ± 6**	**29 ± 5**	**24 ± 5**	**22 ± 5**	**21 ± 5**	**11 ± 3**	**13.6 ± 4.3**	**4.7 ± 1.0**	**2.9 ± 2.2**	**1.5 ± 1.0**	**1.3 ± 1.2**	**0.4 ± 0.7**
V	12** ± **3	1** ± **1	–	–	–	–	10.1** ± **4.0	1.1** ± **1.0	0.2** ± **0.4	–	–	–
D	25** ± **5	**15 ± 4**	**9 ± 3**	4** ± **2	–	–	9.6** ± **3.4	**3.2 ± 2.2**	**1.2 ± 0.9**	0.7** ± **0.7	0.5** ± **0.8	0.1** ± **0.3
T	**38 ± 6**	**24 ± 5**	**17 ± 4**	**6 ± 2**	**4 ± 2**	**4 ± 2**	**9.0 ± 3.5**	**2.3 ± 1.7**	**0.9 ± 1.1**	**0.3 ± 0.4**	**0.2 ± 0.5**	**0.1 ± 0.3**
I	1** ± **1	–	–	–	–	–	2.8** ± **1.5	0.3** ± **0.5	–	–	–	–
H	5** ± **2	5** ± **2	5** ± **2	**5 ± 2**	**4 ± 2**	3** ± **2	2.5** ± **1.3	1.1** ± **0.8	0.9** ± **1.0	**0.9 ± 0.8**	**0.5 ± 0.7**	0.4** ± **0.7
F	4** ± **2	–	–	–	–	–	2.4** ± **2.3	0.3** ± **0.7	–	–	–	–
N	8** ± **3	2** ± **1	–	–	–	–	2.2** ± **1.3	0.5** ± **0.7	–	–	0.1** ± **0.2	–
C	3** ± **2	–	–	–	–	–	1.8** ± **1.6	0.6** ± **0.7	0.4** ± **0.5	0.1** ± **0.2	–	–
W	–	–	–	–	–	–	1.1** ± **0.9	0.1** ± **0.2	–	–	–	–
M	–	–	–	–	–	–	0.3** ± **0.5	–	–	–	–	–
Y	–	–	–	–	–	–	0.1** ± **0.2	–	–	–	–	–

C indicates the number of cases associated with an amino acid motif of length between 4 and 9 (895 cases) and R indicates the average motif counts measured on 20 random extractions from human proteome (each sample contains 895 cases)^37^. The standard deviation associated with 20 extractions is reported. Homo-repeats with standardized Z-score > 5 are given in bold.
