# Supplementary material for: Non-random distribution of homo-repeats: links with biological functions and human diseases
Source: Sci Rep. 2016 Jun 3;6:26941. doi: 10.1038/srep26941 (PMC4891720; doi:10.1038/srep26941)

**Non-random distribution of homo-repeats: links with biological functions and human diseases**

**Michail Yu. Lobanov^1^, Petr Klus^2^, Igor V. Sokolovsky^1^, Gian Gaetano Tartaglia^2,3,4*^ and Oxana V. Galzitskaya^1*^**

^1^ Group of Bioinformatics, Institute of Protein Research, Russian Academy of Sciences, 4 Institutskaya str., Pushchino, Moscow Region, 142290, Russia

^2^ Bioinformatics and Genomics Programme, Centre for Genomic Regulation (CRG), Dr Aiguader 88, 08003 Barcelona, Spain

^3^ Universitat Pompeu Fabra (UPF), 08003 Barcelona, Spain

^4^ Institució Catalana de Recerca i Estudis Avançats (ICREA), 23 Passeig Lluís Companys, 08010 Barcelona, Spain

To whom correspondence should be addressed: OVG: [ogalzit@vega.protres.ru](mailto:ogalzit@vega.protres.ru) and GGT: gian.tartaglia@crg.eu

**Supplementary Table 1. List of 97 eukaryotic and 25 bacterial proteomes used in this work**

| Eukaryota | Fungi | Bacteria*** |
| --- | --- | --- |
| | Metazoa | 25.H_sapiens  22974.B_taurus  59.M_musculus  122.R_norvegicus  21457.G_gallus  20721.D_rerio  22388.T_nigroviridis  17.D_melanogaster  25396.D_pseudoobscura  31436.A_aegypti  78607.A_darlingi  22426.A_gambiae  21633.C_briggsae  9.C_elegans  64800.L_loa  79720.T_spiralis  30565.N_vectensis | | --- | --- | | Viridiplantae | 23214.O_sativa  3.A_thaliana  33157.Micromonas_sp  29351.O_lucimarinus  25972.O_tauri | | Stramenopiles* | 35109.E_siliculosus | | Choanoflagellida^**^ | 30562.M_brevicollis | | Euglenozoa^*^ | 83400.L_braziliensis  83363.L_infantum  71330.T_brucei_gambiense  33602.T_cruzi | | Alveolata^*^ | 32114.P_berghei  31998.P_chabaudi  493.P_falciparum  31342.P_knowlesi  31632.P_vivax  21631.P_yoelii | | Amoebozoa^*^ | 21395.D_discoideum  35301.P_pallidum | | Diplomonadida^*^ | 33600.G_intestinalis_ATCC_50803  35295.G_intestinalis_ATCC_50581  65115.G_intestinalis | | 25591.P_nodorum  79905.P_teres  29154.A_clavatus  33020.A_flavus  22118.N_fumigata_ATCC_MYA-4609  31018.N_fumigata_CEA10  29130.A_niger  23077.A_oryzae  28239.A_terreus  29157.N_fischeri  31898.P_chrysogenum  32999.P_marneffei  33056.T_stipitatus  34218.C_posadasii_C735  34307.P_brasiliensis_Pb03  34389.P_brasiliensis_Pb18  34392.P_brasiliensis_ATCC_MYA-826  34310.A_capsulata_ATCC_26029  34967.A_capsulata_H143  34495.A_dermatitidis_SLH14081  34498.A_dermatitidis_ER-3  35919.A_benhamiae  34471.A_otae  35921.T_verrucosum  34386.U_reesii  30100.B_fuckeliana  30103.S_sclerotiorum  22024.C_albicans_SC5314  32738.C_dubliniensis  19665.C_glabrata  34491.C_tropicalis  20018.D_hansenii  29447.L_elongisporus  29448.M_guilliermondii  28727.S_stipitis  20011.Y_lipolytica  34493.C_lusitaniae  34482.L_thermotolerans  30091.S_cerevisiae_YJM789  31651.S_cerevisiae_RM11-1a  34506.S_cerevisiae_JAY291  35062.S_cerevisiae_Lalvin_EC1118  71242.S_cerevisiae_ATCC_204508  30097.V_polyspora  79902.C_graminicola  35359.V_albo-atrum  34970.N_haematococca  22028.M_oryzae  25585.C_globosum_NBRC_6347  22025.N_crassa  35280.S_macrospora  79908.P_graminis  31020.C_cinerea  31023.L_bicolor  33031.P_placenta  20846.C_neoformans_JEC21  21380.C_neoformans_B-3501A  22029.U_maydis | | Acidobacteria | 25797.S_usitatus | | --- | --- | | Actinobacteria | 33926.C_acidiphila  35278.Frankia_sp_EuI1c  35534.F_sp_EUN1f  33113.R_opacus  25456.Rhodococcus_sp  37022.A_mediterranei  74443.K_setae  131.S_avermitilis  36666.S_bingchenggensis  84.S_coelicolor  34910.S_scabies  58962.S_violaceusniger  34011.S_roseum | | Proteobacteria | 112.B_japonicum  22343.Burkholderia_sp_ATCC_17760  25388.B_xenovorans  33616.S_aurantiaca  33223.H_ochraceum  23351.M_xanthus  32044.P_pacifica  30295.S_cellulosum | | Bacteroidetes | 33930.C_pinensis  32144.M_marina | | Chloroflexi | 36622.K_racemifer | |

^*^ Category without rank is given.

^**^The name of order is given because the highest ranks are missing in the taxonomic description.

^***^The superkingdom of bacteria is divided in phyla rather than kingdoms.

**Supplementary Figure 1.** *Amino acids frequencies* for (A) bacterial, eukaryotic (blue rectangles, 122 organisms) and human (white rectangles) proteomes; (B) 6 different kingdoms of eukaryotic proteomes.

**
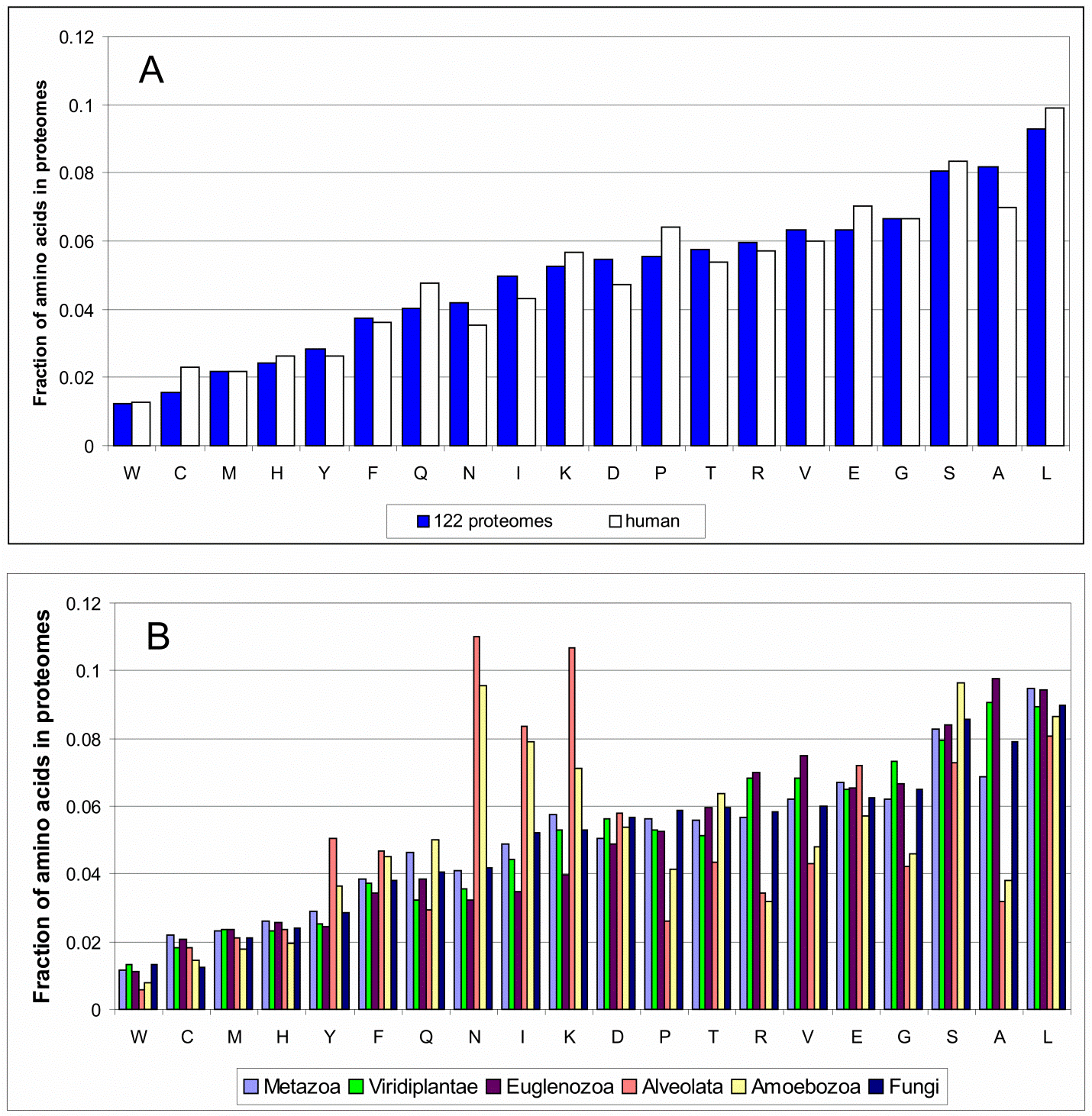
**

**Supplementary data.** *Theoretical vs observed homo-repeat frequencies for all 20 amino acids in 122 proteomes.*


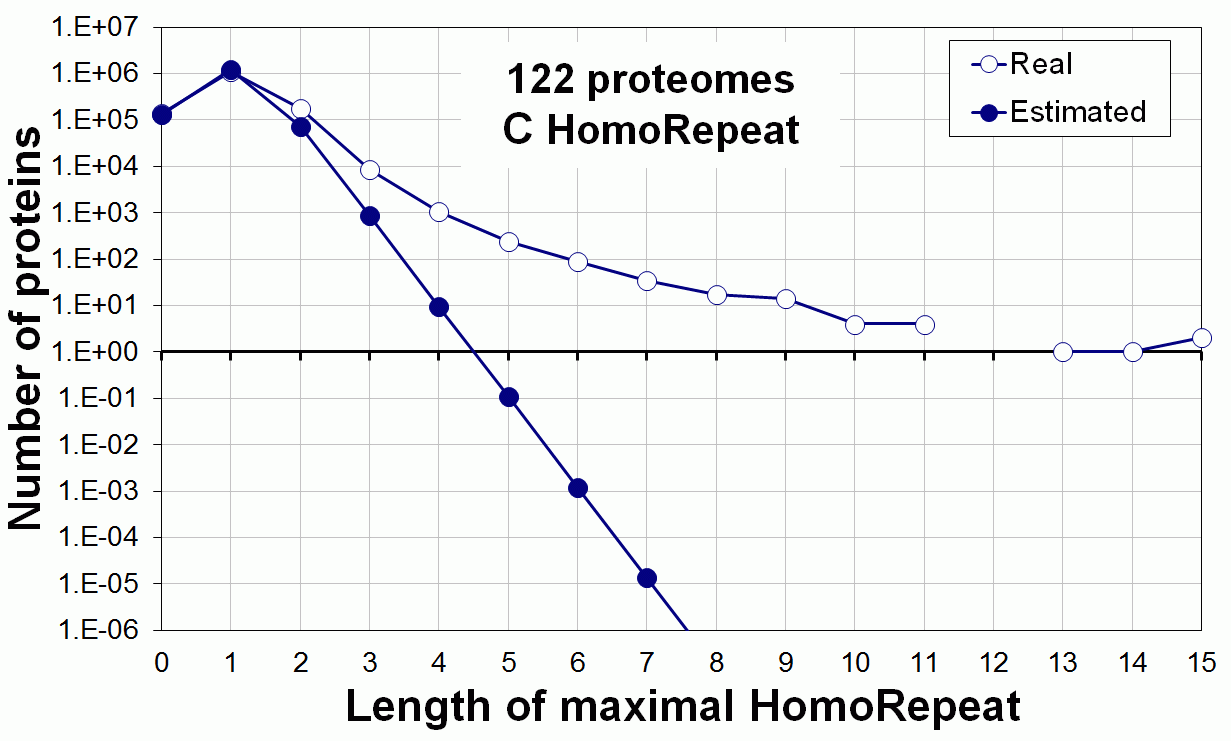


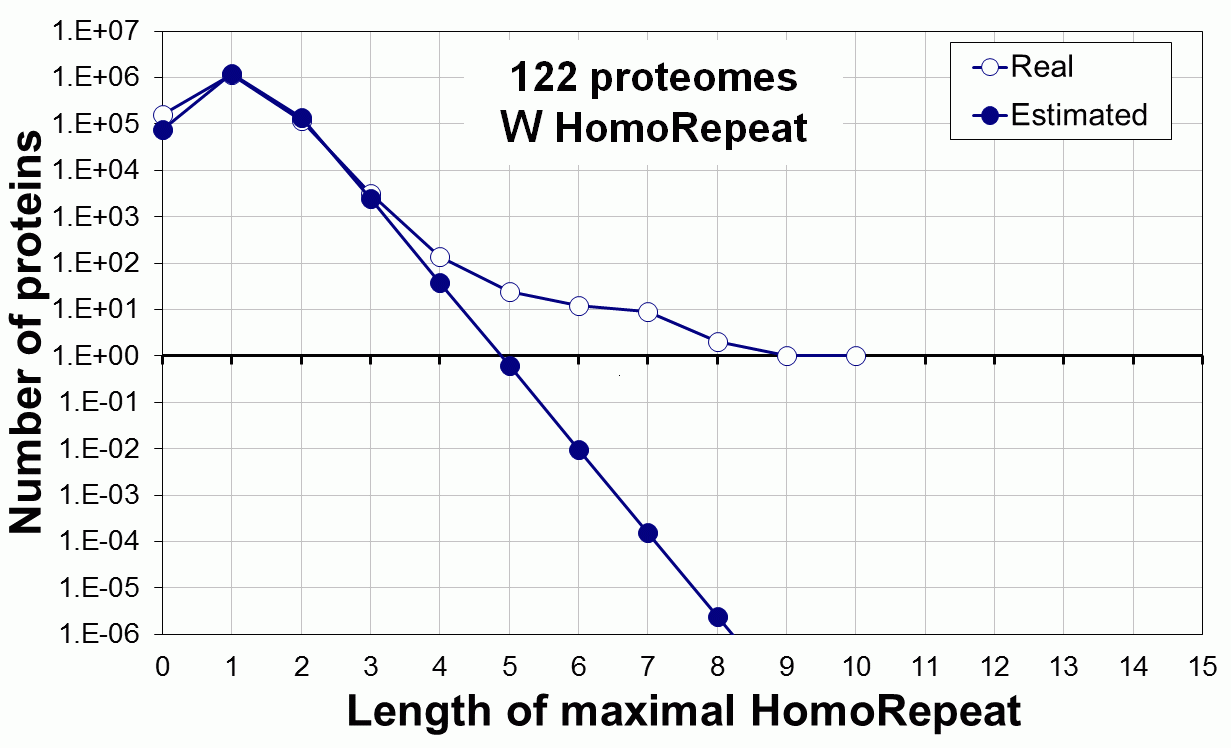

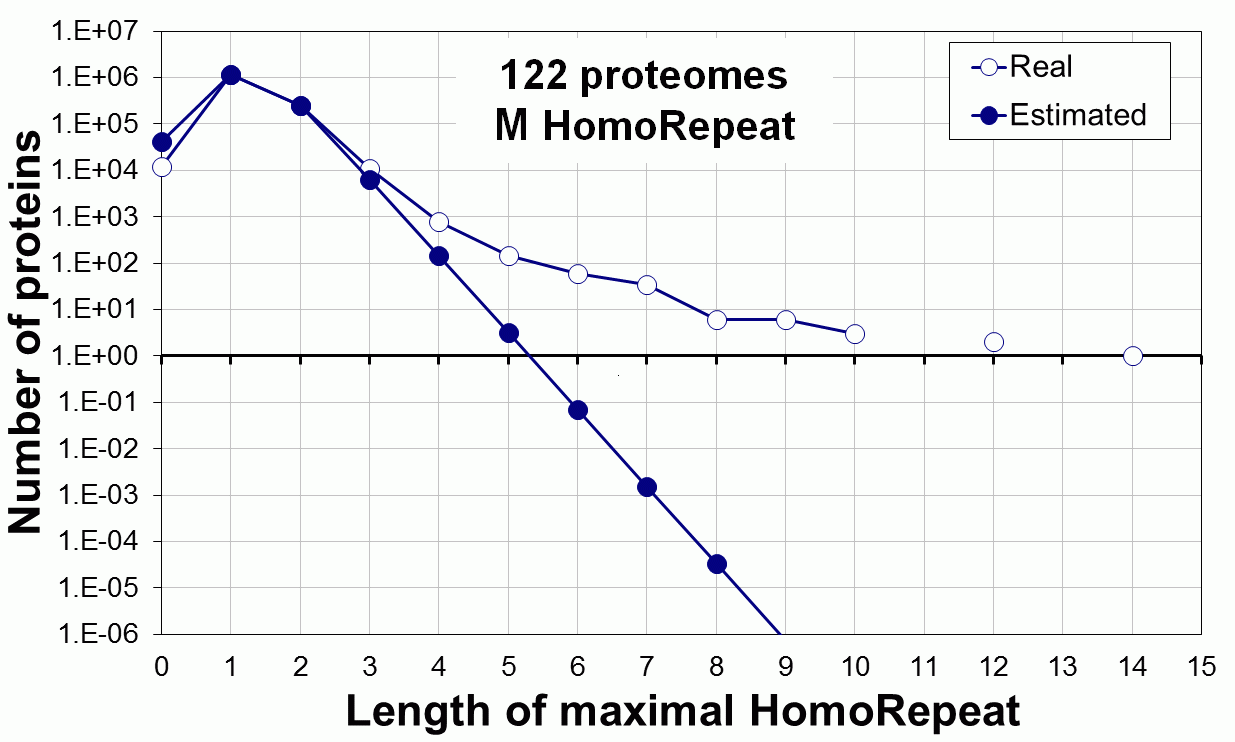

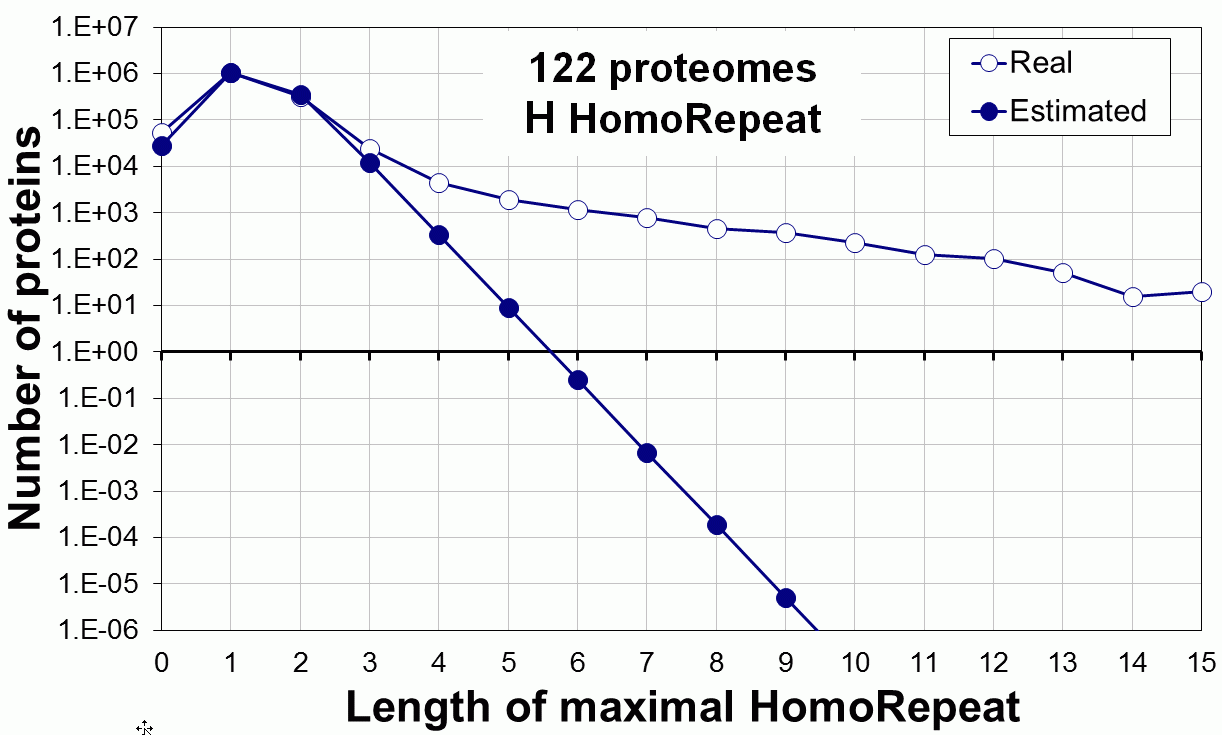


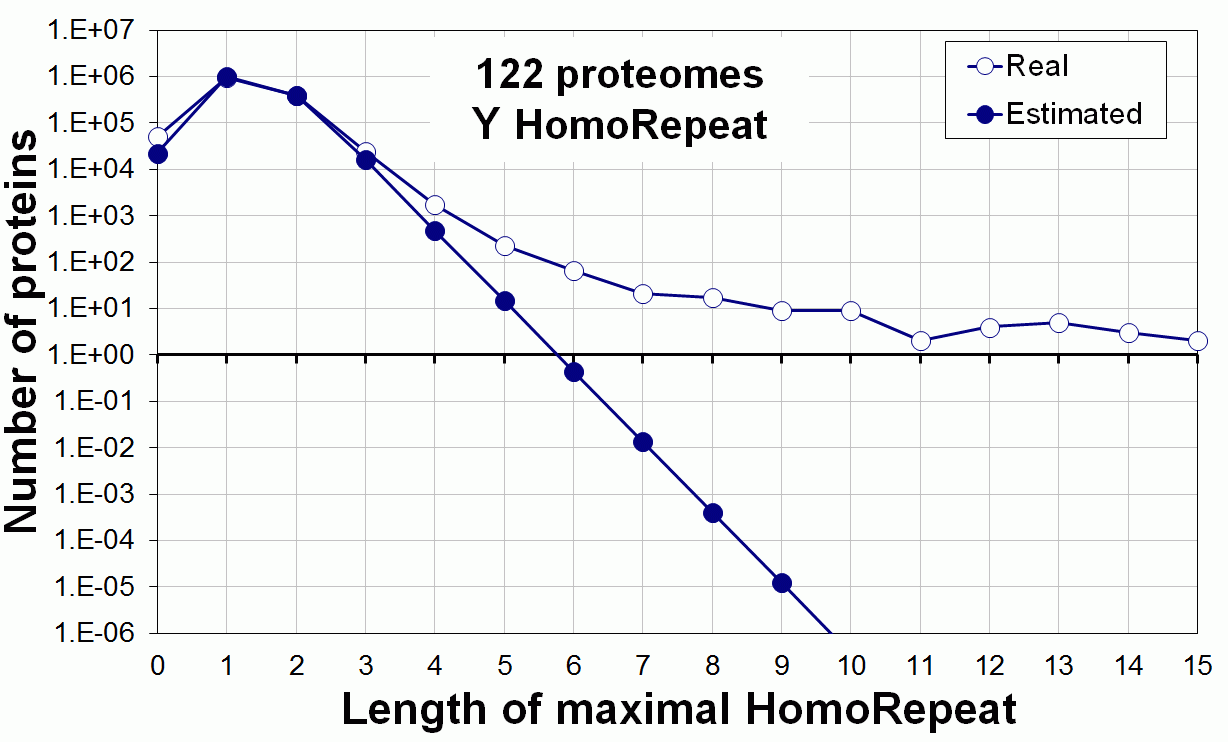

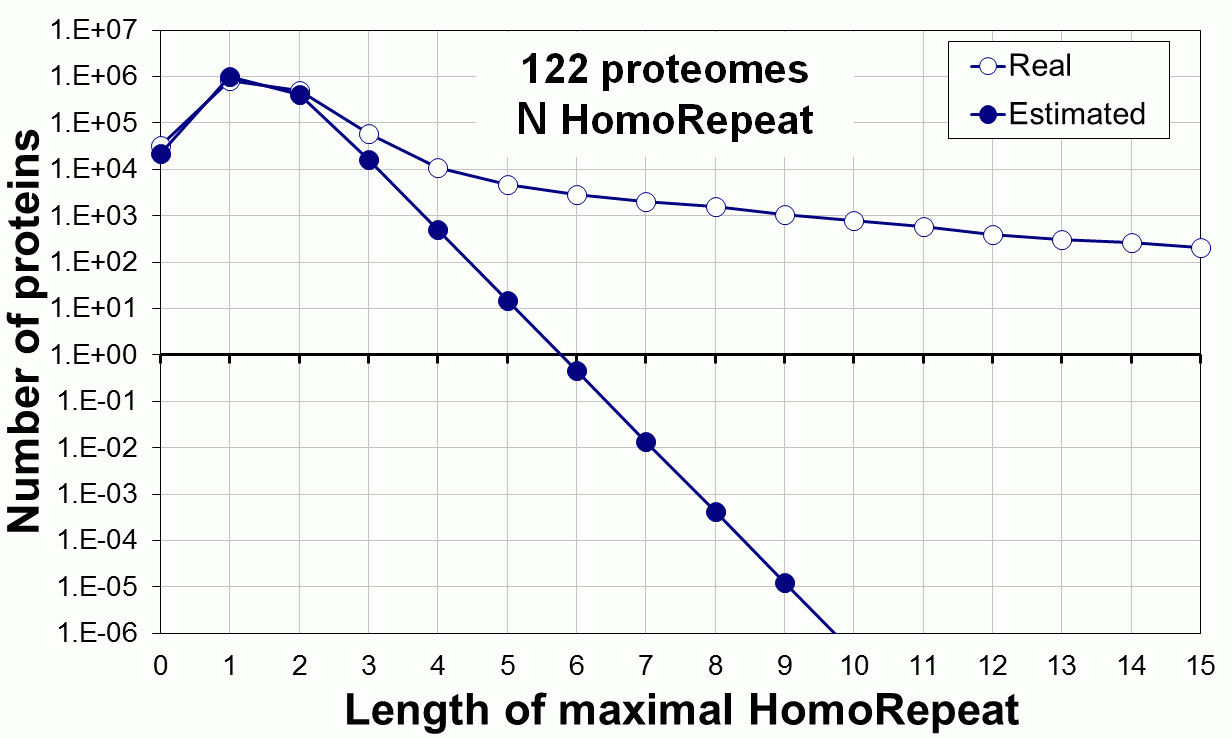


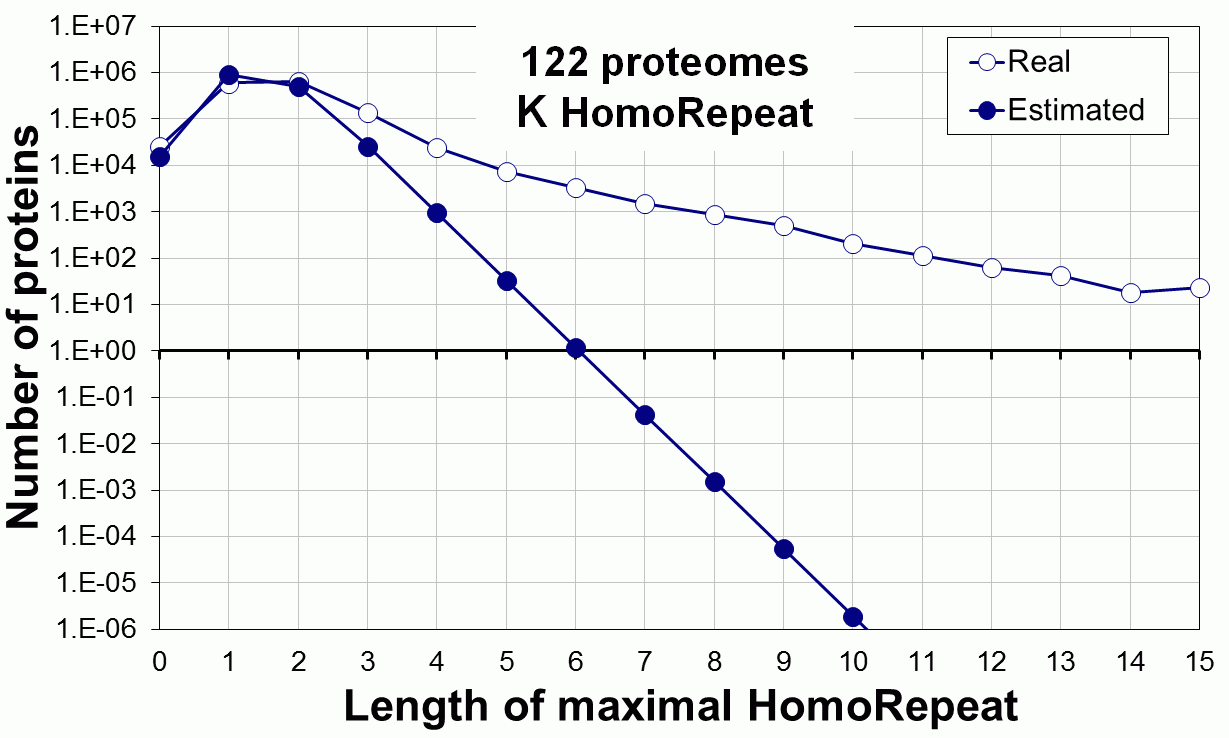

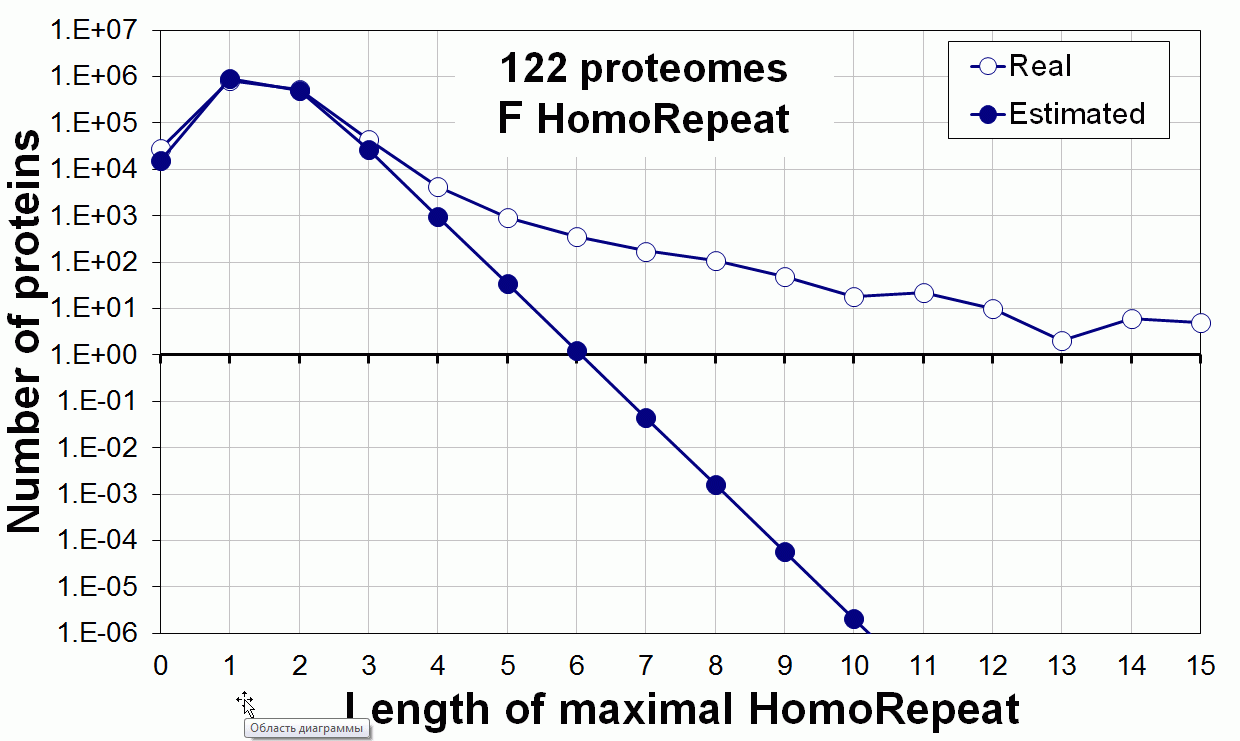

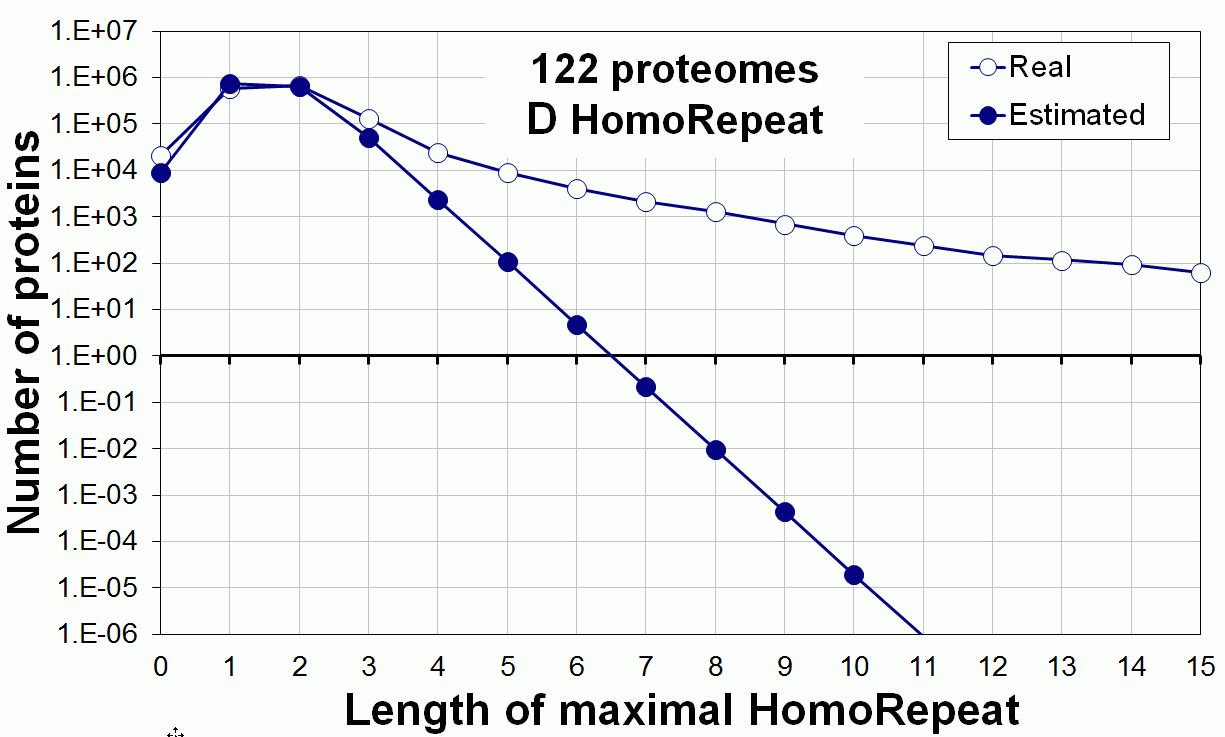

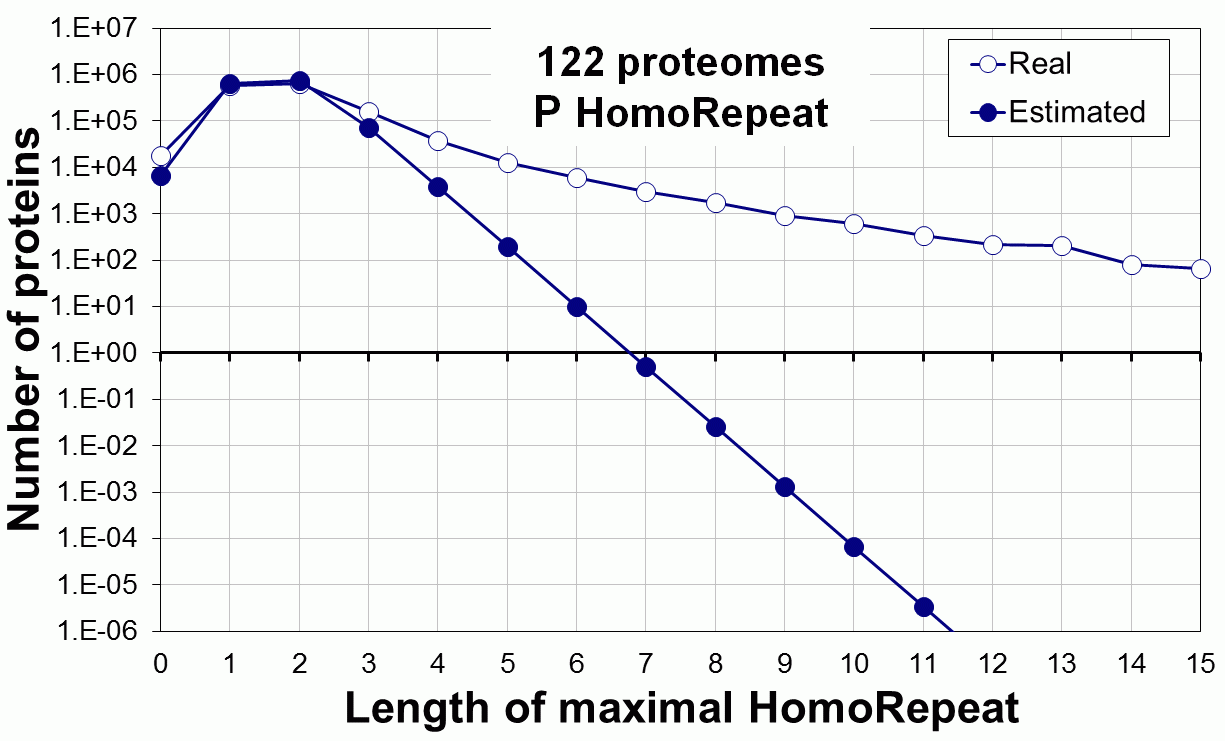

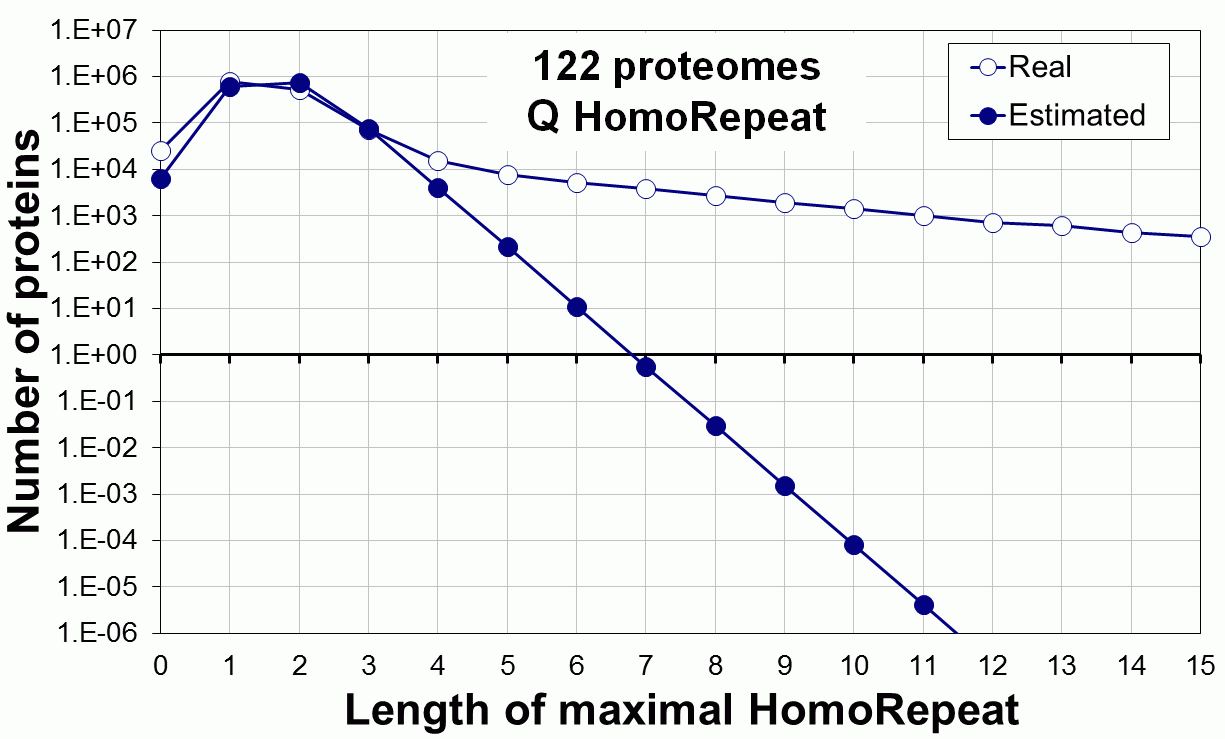

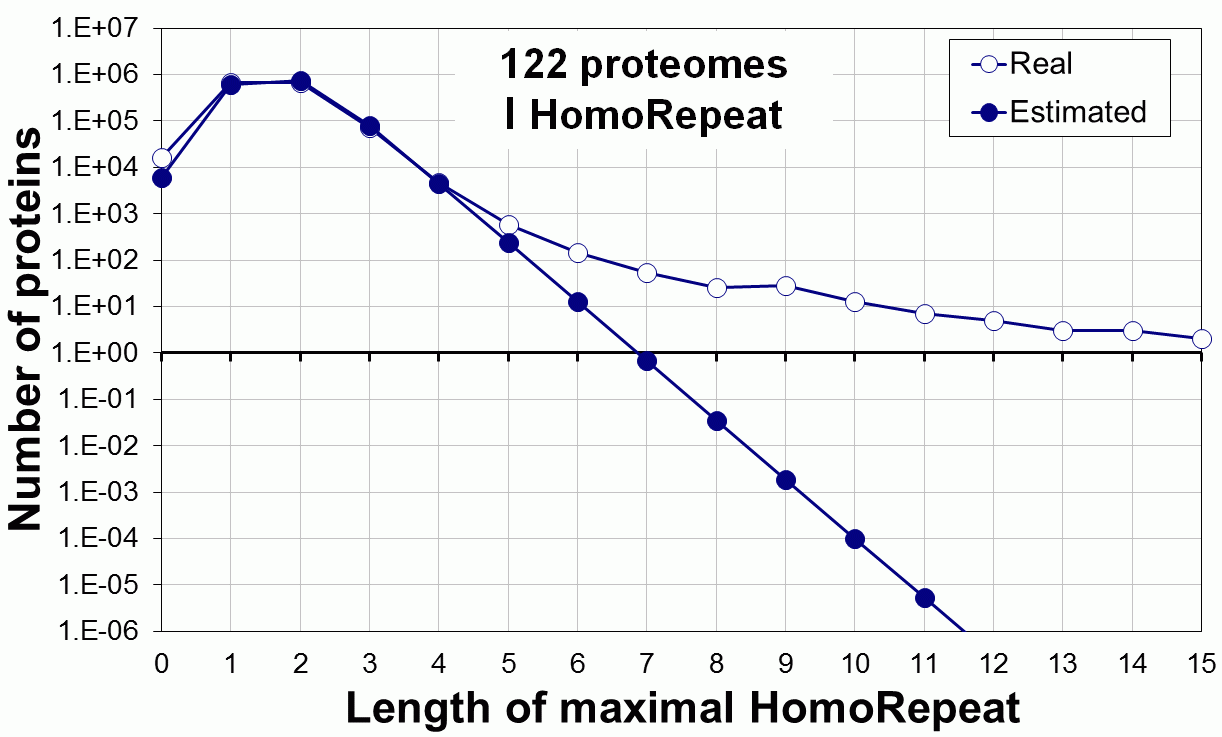

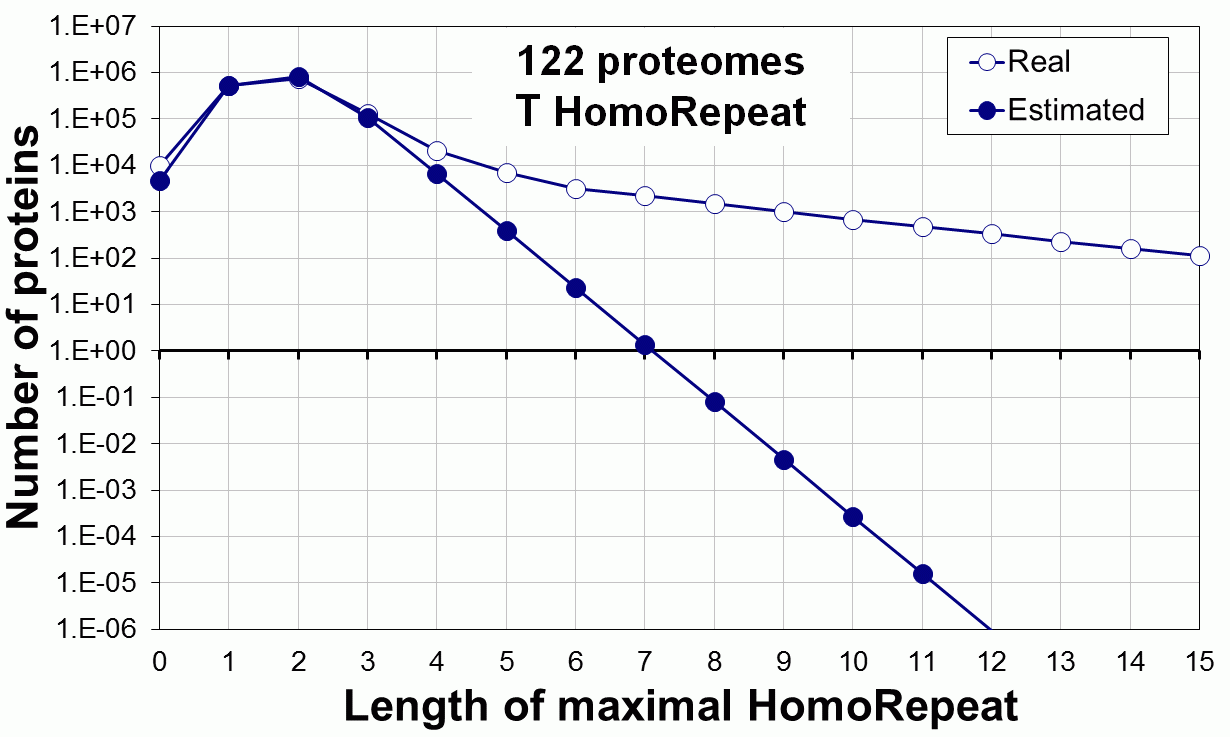

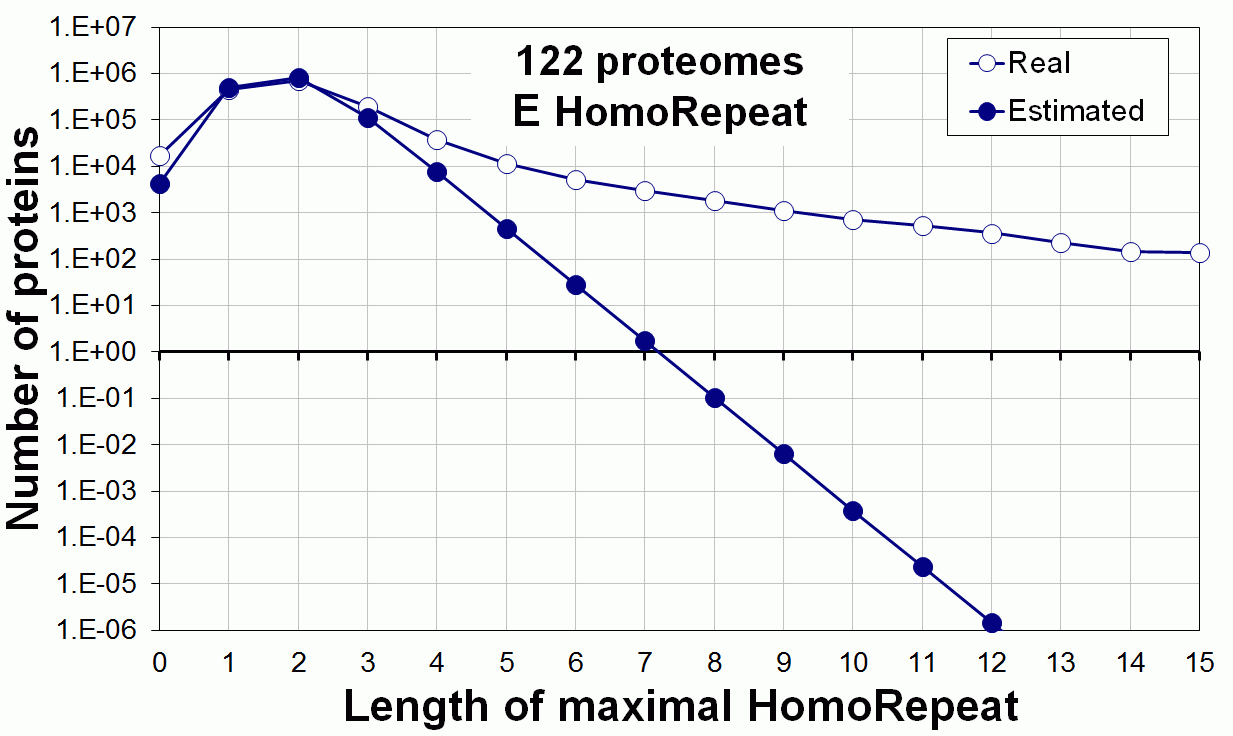

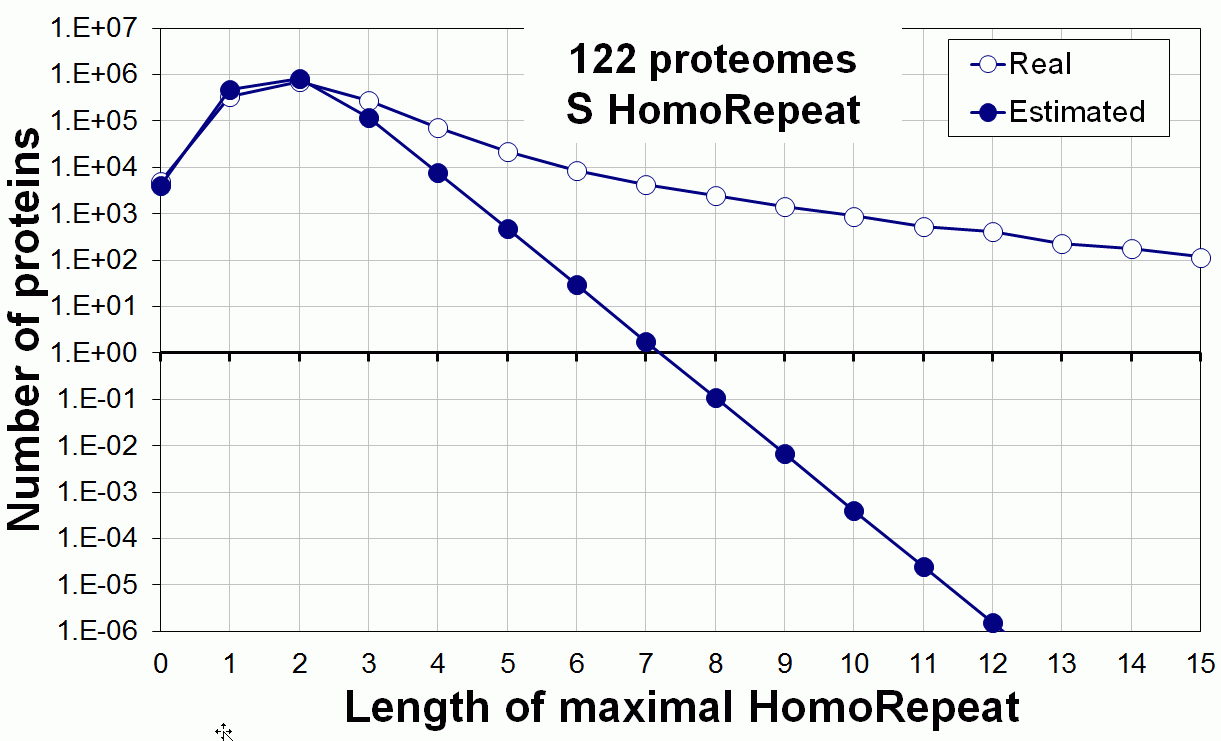

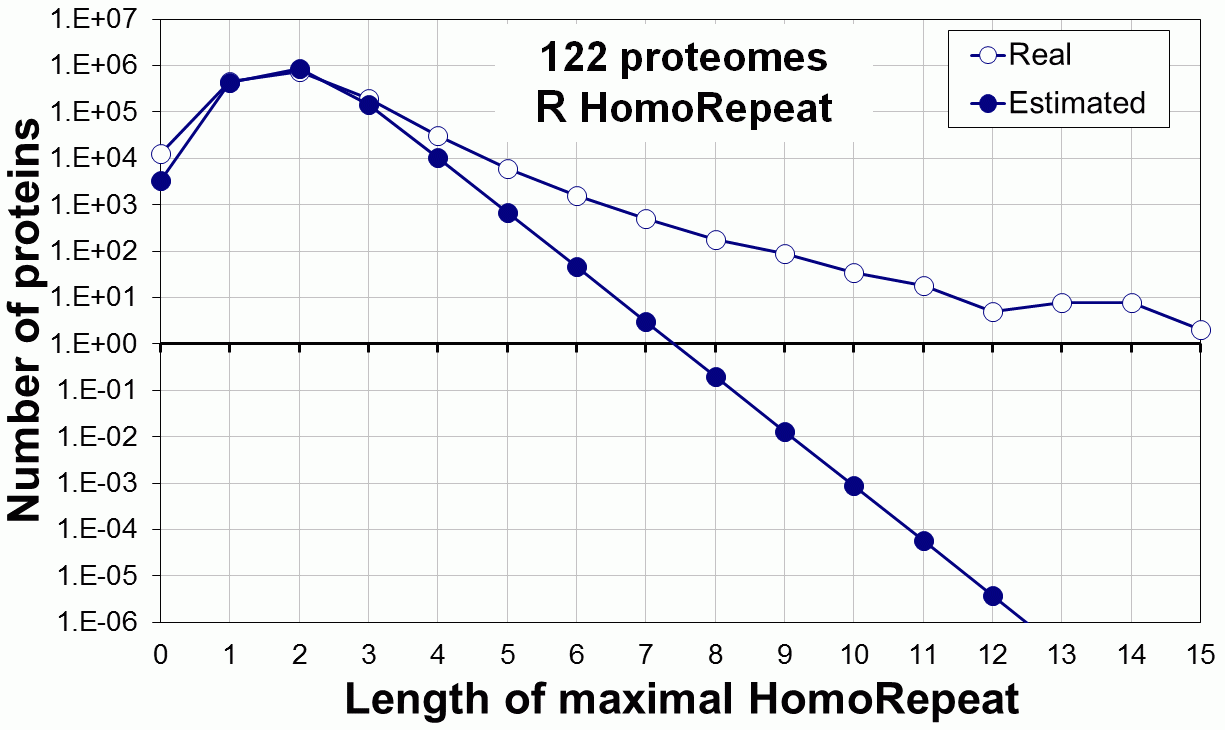

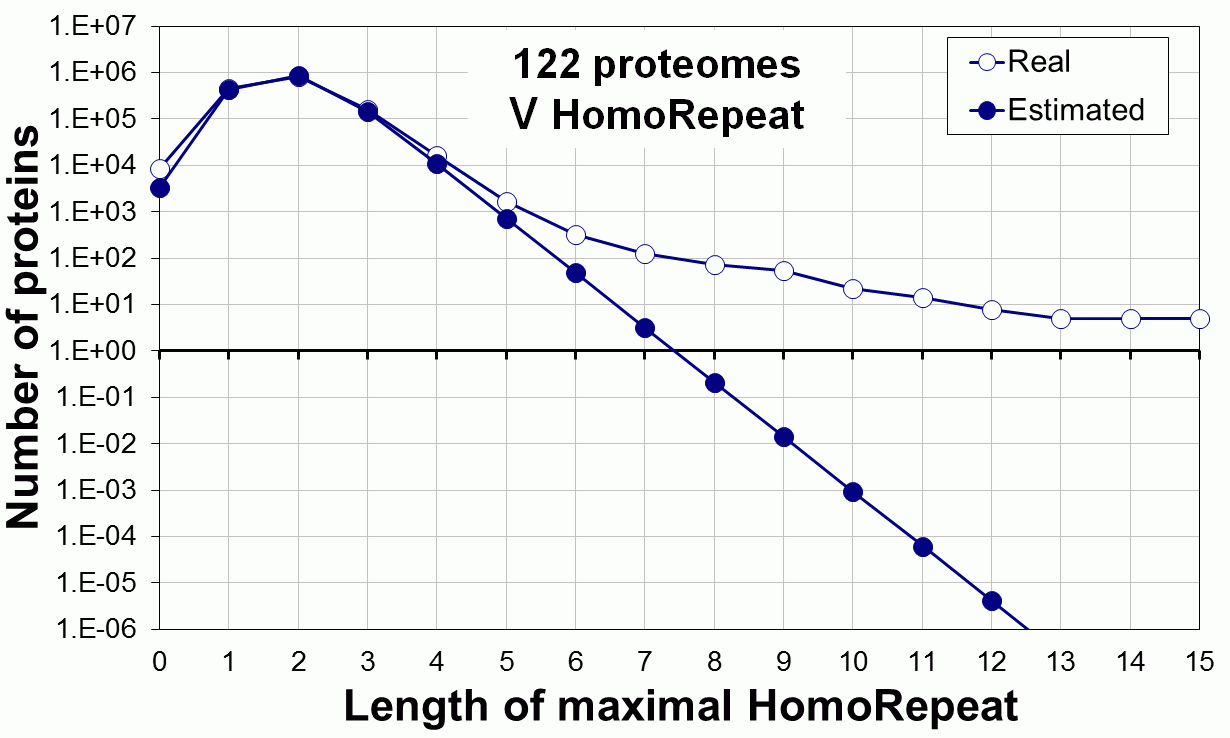

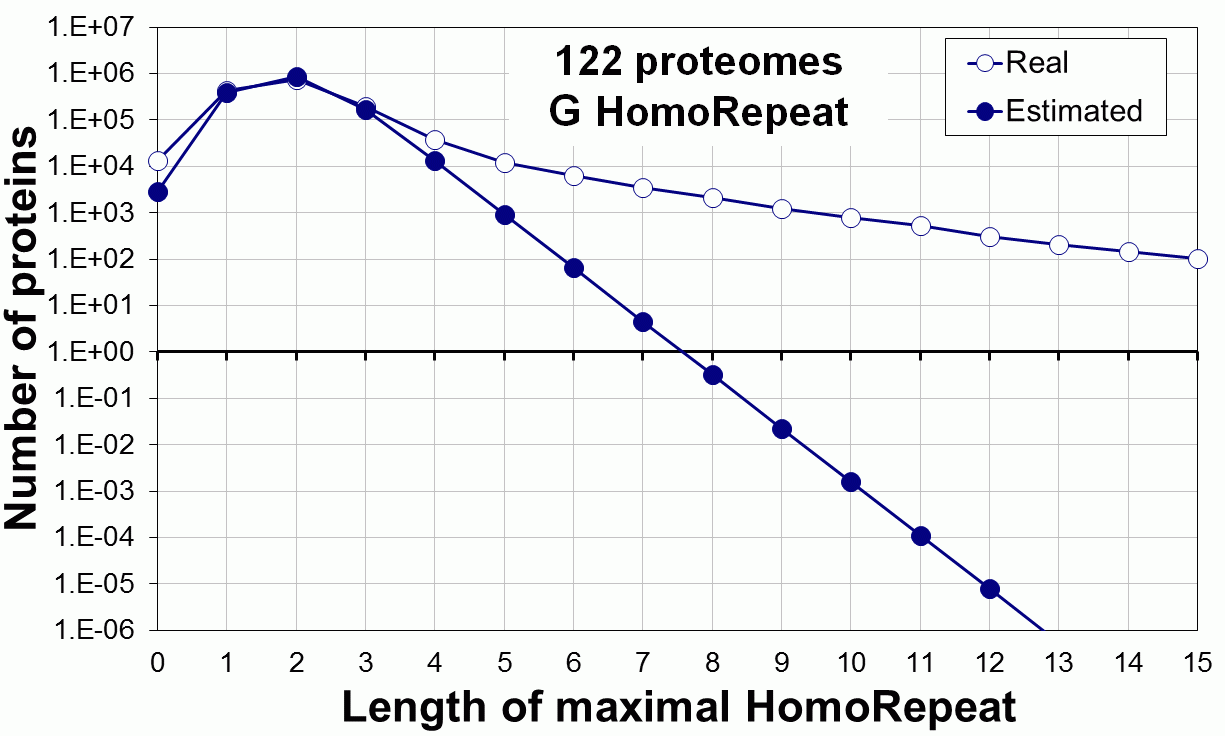

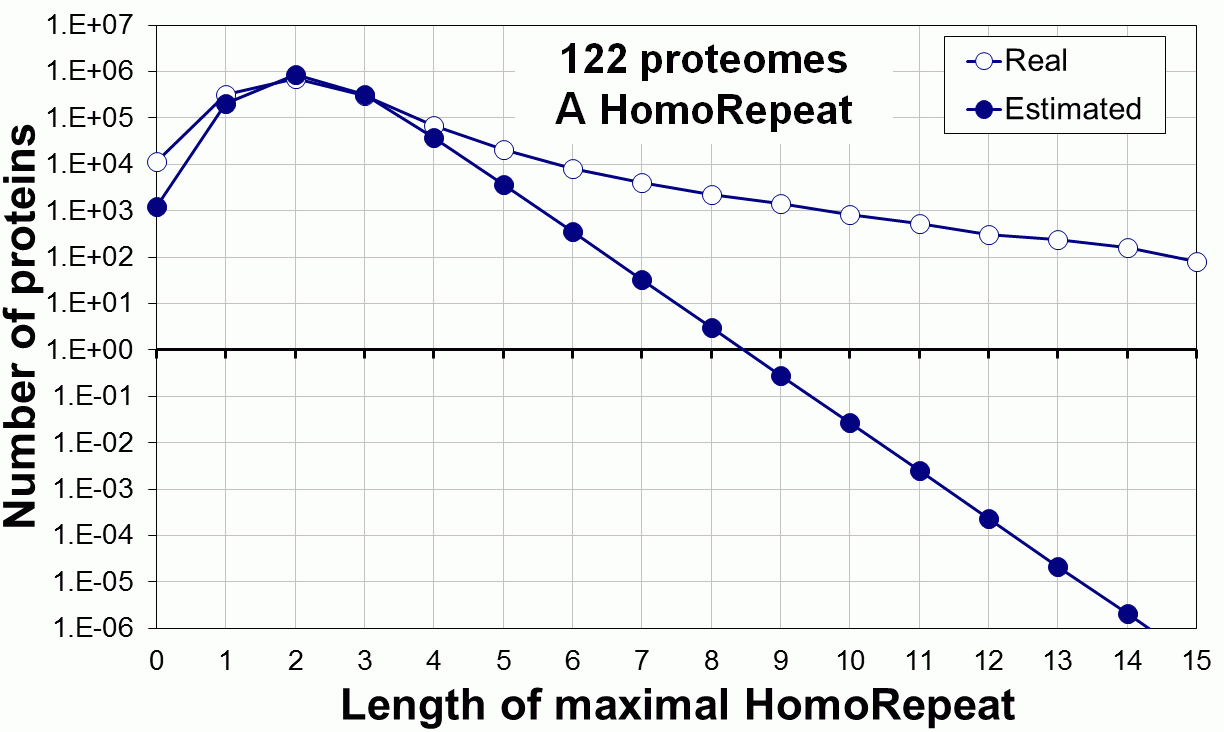

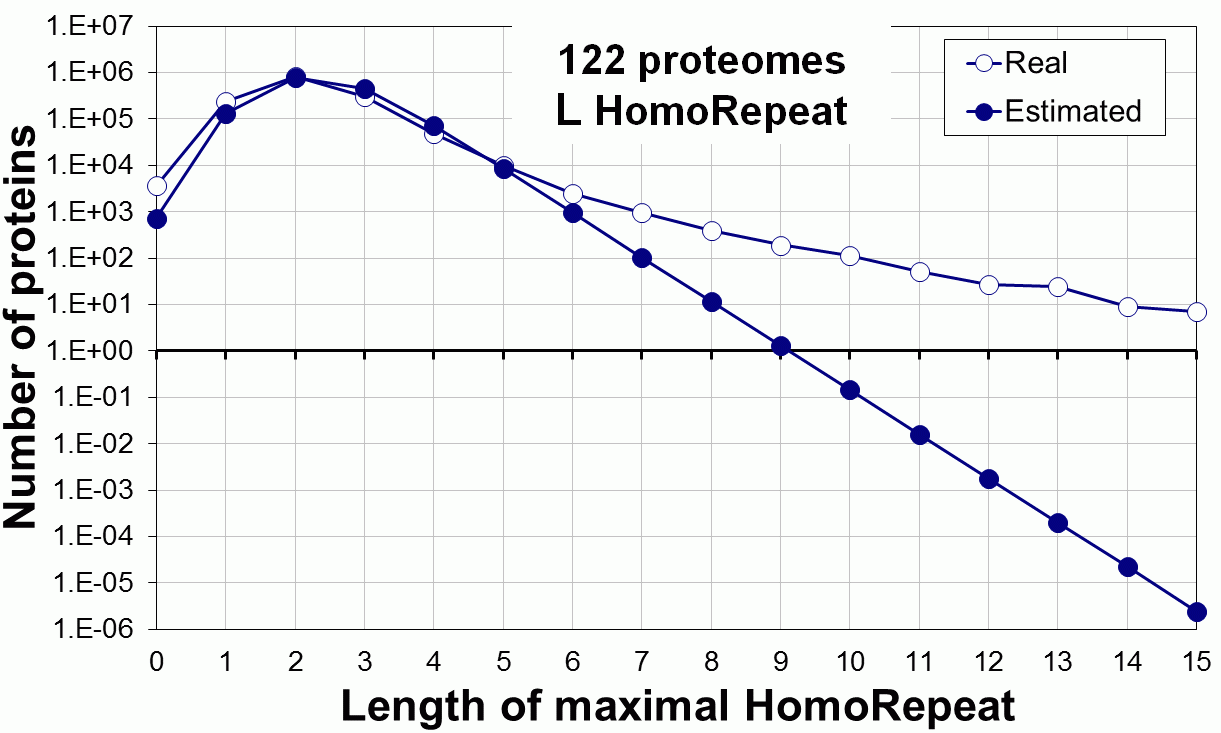

Supplement: Supplementary Information [file srep26941-s1.doc]
